# The aryl hydrocarbon receptor: structure, signaling, physiology and pathology

**DOI:** 10.1038/s41392-025-02500-8

**Published:** 2026-01-16

**Authors:** Xavier Coumoul, Robert Barouki, Charlotte Esser, Thomas Haarmann-Stemmann, B. Paige Lawrence, Jörg Lehmann, Pedro Moura-Alves, Iain A. Murray, Christiane A. Opitz, Gary H. Perdew, William Bourguet

**Affiliations:** 1 Université Paris Cité, Inserm, Health & Functional Exposomics - HealthFex, Paris, France; 2 IUF—Leibniz Research Institute for Environmental Medicine, Düsseldorf, Germany; 3 Department of Environmental Medicine and Department of Microbiology and Immunology, University of Rochester, Rochester, NY USA; 4 Department of Preclinical Development and Validation, Fraunhofer Institute for Cell Therapy and Immunology IZI, Leipzig, Germany; 5 Fraunhofer Cluster of Excellence Immune-Mediated Diseases CIMD, Leipzig, Germany; 6 i3S, Instituto de Investigação e Inovação em Saúde, Universidade do Porto, Porto, Portugal; 7 IBMC, Instituto de Biologia Molecular e Celular, Universidade do Porto, Porto, Portugal; 8 Department of Veterinary and Biomedical Sciences, Center for Molecular Toxicology and Carcinogenesis, The Pennsylvania State University, University Park, PA USA; 9 German Cancer Research Center (DKFZ), Division of Metabolic Crosstalk in Cancer and the German Cancer Consortium (DKTK), DKFZ Core Center Heidelberg, Heidelberg, Germany; 10 Center for Structural Biology, University Montpellier, Inserm, CNRS, Montpellier, France

**Keywords:** Toxicology, Physiology, Cancer, Immunology, Cell biology

## Abstract

The aryl hydrocarbon receptor (AHR) is a ligand-dependent transcription factor of the bHLH/PAS protein family. In this review, we explore the multifaceted roles of AHR in both health and disease, tracing its recognition as a xenobiotic sensor and a central regulator of physiological homeostasis. We begin by recounting six decades of discoveries that have shaped our understanding of AHR, from its canonical function in environmental sensing to its critical roles in development, immune regulation, barrier tissue integrity, and host–microbe interactions. We detail recent structural breakthroughs that have illuminated the ligand-binding mechanisms and regulation of AHR, providing key insights into its activation and transcriptional control. We also highlight the diversity of AHR ligands, ranging from environmental toxins to microbial and dietary metabolites of tryptophan, and their context-dependent effects on AHR activation through the canonical pathway and noncanonical signaling mechanisms. We examine the involvement of AHR in pathologies such as cancer and autoimmune and inflammatory diseases and its potential as a therapeutic target. Finally, emphasis is placed on recent advances and future developments in drug design, aiming to develop modulators with clinical efficacy. This comprehensive synthesis underscores the dual role of AHR as a master integrator of both environmental and endogenous cues. Placing AHR within broader frameworks, such as the exposome, opens new avenues for therapeutic innovation and more effective strategies for disease prevention.

## Introduction

**Fig. 1:**
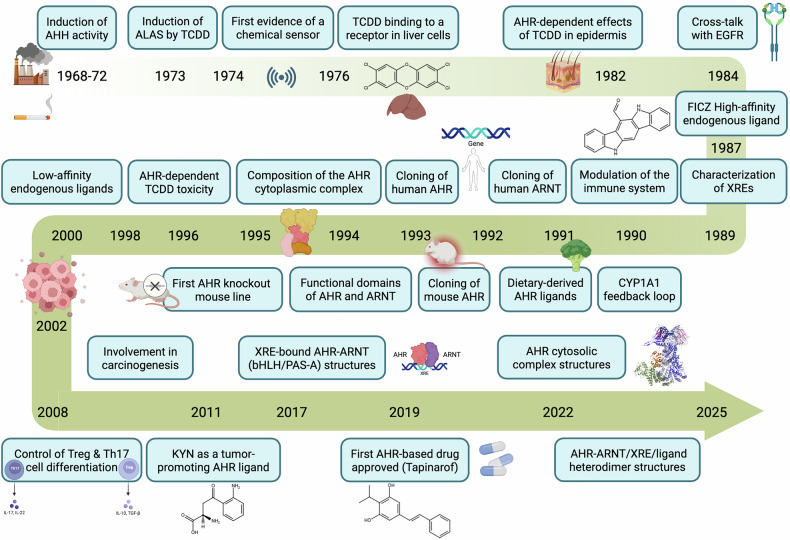
Timeline of major advancements in AHR studies. The figure was created via BioRender

The story of the aryl hydrocarbon receptor (AHR) spans over six decades, marked by pivotal discoveries that have transformed our understanding of its roles in toxicology, physiology, and disease (Fig. 1). The story begins in the early 1970s with the discovery of its role in xenobiotic metabolism. Before this decade, early studies identified enzyme induction by polycyclic aromatic hydrocarbons (PAHs), such as aryl hydrocarbon hydroxylase (AHH), in the rat liver and gastrointestinal (GI) tract. Genetic differences in PAH-induced AHH activity between mouse strains suggested heritable regulation.^1^ In 1974, researchers first reported evidence of AHR as a “sensor” of foreign chemicals, noting its ability to upregulate cytochrome P450 family 1 (CYP1) and other enzymes that typically metabolize chemicals.^2^ In fact, the manuscript was sent for review in 1973 but was first rejected for “heresy”.^1^ The concept that xenobiotics might regulate an intracellular protein appeared implausible at that time. A significant milestone was reached in 1976 when it was demonstrated that 2,3,7,8-tetrachlorodibenzo-p-dioxin (TCDD), a byproduct of various combustion processes, including forest fires, and a contaminant found in several substances, such as the herbicide agent orange, can exert its effects by binding to a specific cellular component in mouse liver cells.^3^ This binding was found to be saturable and high-affinity, which is consistent with a receptor-mediated mechanism. This finding was crucial for understanding how cells may be affected by this chemical and laid the groundwork for future AHR research.

Among the early pioneers, both Alan Poland and Daniel Nebert, two physician scientists, played foundational roles in shaping the field of AHR research. Nebert introduced an evolutionary and developmental biology perspective,^4^ whereas Poland, with a strong background in receptor pharmacology, provided experimental evidence for the physiological relevance of AHR beyond xenobiotic metabolism. Notably, Poland’s landmark Science paper demonstrated the induction of δ-aminolevulinic acid synthetase (ALAS) by TCDD, directly implicating AHR in heme biosynthesis.^5^ The effects of AHR ligands in the murine epidermis and on the differentiation of the keratinizing XB cell line were subsequently demonstrated in 1982 and 1984, respectively.^6,7^ These studies helped recognize the broader role of AHR in regulating cell growth and differentiation^8^ and establish the stage for the physiological importance of this receptor.^9^ In addition, the discovery of crosstalk between AHR and other signaling pathways, such as the EGF receptor, underscored the concept that the receptor plays an integrative role in cellular signaling networks.^10^ The recognition of dietary-derived ligands as endogenous AHR modulators also dates back to the early 1990s.^11^ These foundational studies helped establish AHR as a multifaceted regulator of both toxicological and physiological processes.

Between 1973 and 1989, the mouse [Ah] locus was shown to be relevant to chemical carcinogenesis, mutagenesis, toxicity, and teratogenesis,^1^ marking the beginning of understanding the role of AHR in a variety of physiological processes beyond xenobiotic metabolism. Inflammation was among the first processes suspected to be regulated by AHR. The molecular characterization of AHR advanced with the cloning of its partner ARNT (AHR Nuclear Translocator) in 1991,^12^ followed by stepwise cloning of the mouse AHR itself, first through N-terminal fragment and protein characterization, and then the gene.^13–16^ The human AHR cDNA^17^ and gene^18^ were subsequently identified between 1993 and 1994. This was followed by another significant milestone in 1995, when the first *Ahr*^-/-^ knockout mouse line was reported.^19,20^ Other knockouts further highlighted the complexity of the AHR-ARNT system, with phenotypes sometimes slightly different.^21,22^ These developments provided powerful tools for studying receptor functions in vivo. Crucially, the work on AHR-regulated gene transcription and the characterization of xenobiotic response elements (XREs) have provided essential mechanistic insights into AHR signaling.^23–25^ In addition, early and sustained investigations into the regulation of AHR ligands by CYP1A1 established the concept of a negative feedback loop, a key regulatory mechanism in AHR biology.^26^

The molecular cloning of AHR and its dimerization partner ARNT in the early 1990s was also a crucial step in the development of a basic model of AHR signal transduction.^27–29^ These experiments revealed that both AHR and ARNT are structurally related, heterodimeric partners harboring both basic helix-loop-helix (bHLH) and PER-ARNT-SIM (PAS) homology domains within their N-terminal halves.^30,31^ In the 1990s, the subunit composition of the AHR cytoplasmic core complex was established as AHR, the chaperone heat-shock protein 90 (Hsp90) dimer and the cochaperone X-associated protein 2 (XAP2).^32–34^ By the early 2000s, it became clear that AHR plays essential roles in maintaining homeostasis, with its domain structures and functions being conserved across evolution and commonly observed in nearly all multicellular organisms.

The ability of AHR ligands to modulate the immune system, including effects on T-cell differentiation, was discovered in the early 1990s.^35–37^ A major shift in AHR research occurred in 2008 when two independent manuscripts^38,39^ reported that different AHR ligands could increase regulatory T-cell (Treg) generation, whereas others promote T helper 17 (Th17) cell differentiation both in vitro and in vivo. This discovery sparked intense interest among immunologists and researchers studying autoimmunity.

Low-affinity tetrapyrroles and lipid-derived molecules, such as bilirubin, a heme breakdown product that modulates oxidative stress and inflammation, or arachidonic acid metabolites that regulate inflammation and immune responses, were identified in the late 1990s as potential endogenous AHR ligands.^40,41^ However, breakthroughs have been made in the identification of tryptophan metabolites that act as high-affinity endogenous ligands of AHR, such as formylindolo[3,2-b]carbazole (FICZ),^42–44^ or molecules that while acting as weak agonists, such as kynurenine, may be present at high concentrations in cells.^45^ Identifying kynurenine as an endogenous AHR ligand has shed light on the role of the receptor in immune regulation and the indoleamine 2,3-dioxygenase-1 (IDO1)-AHR pathway.^45^ In addition, some microbe-derived indoles (e.g., indole-3-acetic acid, indirubin, and indigo) have been found to activate AHR and may influence gut immunity and barrier functions.^46,47^ Although much of the research on the barrier function of AHR has focused on the intestine, especially concerning immune responses, studies from the 2010s revealed that AHR is also widely expressed in other barrier tissues, such as the skin^48^ and lungs.^49^ In the skin, it appears to play sensory and protective roles, including the formation of tight junctions and the production of antimicrobial agents such as defensins.^48,50,51^

Since 2010, disruptions in AHR signaling have been shown to be involved in the development of diseases such as inflammatory bowel disease (IBD) and atopic dermatitis (AD). AHR has since emerged as a key sensor of the chemical exposome, including a wide range of natural molecules, and a regulator of various physiological and pathophysiological processes.^52,53^ Naturally, the development of modulators of AHR function has advanced significantly. After decades of effort, the structure of AHR has been elucidated, opening new avenues for understanding the effects of both xenobiotic and endogenous ligands and enabling more rational approaches to drug design.^54–57^ There is growing interest in developing targeted therapies that modulate AHR activity, potentially opening new avenues for medical intervention in autoimmune diseases such as rheumatoid arthritis,^58^ chronic inflammatory skin disorders^59^ and cardiovascular and pulmonary diseases.^60^ However, it is worth noting that, to date, tapinarof remains the only AHR ligand approved as a therapeutic drug, first in China in 2019 and subsequently in the United States in 2022, for the treatment of psoriasis and AD (see the “AHR-targeting therapies” section for greater details and references). The involvement of AHR in cancer, which has long been suspected due to its connection with CYP1 enzymes in PAH metabolism, has emerged as more complex and multifaceted. On the one hand, AHR can inhibit tumor growth by influencing immune responses and metabolic pathways; on the other hand, AHR has become a target for combating tumor immune evasion.^61^

The goal of this comprehensive review is to summarize the latest advances in our understanding of AHR structure, signaling pathways, and physiological functions, as well as the regulation of AHR activity. It also covers the role of AHR in the development of pathological conditions and in regulating the interactions of the host with its chemical environment. This information is crucial for structure- or mechanism-based drug design and the development of AHR-targeting small molecules, which show promise for treating various diseases.

## AHR signaling pathways and crosstalk

The biological outcome of AHR signaling is largely determined by the nature of its bound ligands. Owing to their compound-specific structural and chemical properties, different ligands may cause different conformational changes,^56^ ultimately controlling the AHR by affecting gene transcription or side-tracking AHR into other signaling pathways.

### Canonical AHR pathway

**Fig. 2:**
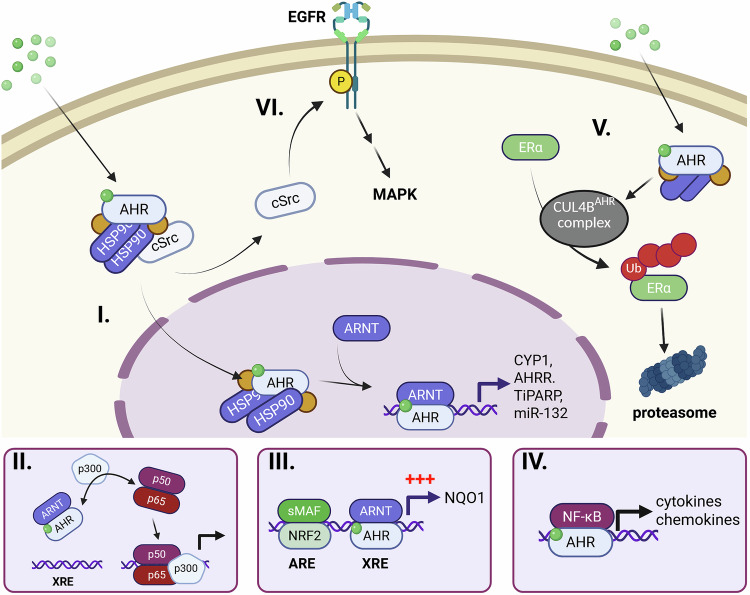
Scheme of various AHR-mediated signaling activities in cells. **I**. In the canonical pathway, the cytosolic AHR complex translocates to the nucleus, dimerizes with ARNT, and initiates target gene expression by binding to xenobiotic response elements. **II**. Transrepression in the nucleus by competition for cofactors, such as p300, which are also used by other transcription factors. **III**. Modulation of chromatin accessibility can increase transcriptional activity (indicated by +++). **IV**. AHR can bind to molecules other than ARNT, such as components of NF-κB. This results in the transcription of yet another set of target genes. **V**. AHR is involved in protein degradation via its ubiquitin ligase function in the cytoplasm. **VI**. At the cell membrane, c-Src released from the AHR complex upon ligand binding can effectively activate EGFR and downstream MAPK signal transduction. For details and references, see the text. The figure was created via BioRender

The primary mode of action of AHR as a transcription factor, often called the “canonical pathway”, is initiated by small-molecular-weight compounds (“ligands”) binding to the ligand-binding domain of AHR and has been extensively investigated, as outlined in several excellent overview articles.^61–63^ Briefly, in the absence of a ligand, AHR is localized in the cytoplasm in a complex with two Hsp90 molecules, XAP2 (also referred to as AHR-interacting protein, AIP), and cochaperone p23, which prevent AHR from translocating into the nucleus and keeping it in a ligand‒affine conformation. In addition, the association of the soluble tyrosine kinase c-Src with this cytoplasmic multiprotein complex is discussed.^64,65^ Upon ligand binding, AHR experiences conformational changes, resulting in the exhibition of a nuclear localization signal and its active transport across the nuclear membrane by the importin machinery.^66^ The tyrosine kinase c-Src may dissociate from the complex during this process and seems to remain in the cytoplasm. In the nucleus, AHR heterodimerizes with its partner protein ARNT and binds to xenobiotic-responsive elements (XREs) in the enhancer/promoter regions of target genes. The DNA-bound AHR-ARNT complex subsequently recruits components of the general transcription machinery, resulting in the RNA polymerase II-mediated transcription of hundreds of target genes (Fig. 2). The canonical pathway can also induce noncoding RNAs, particularly microRNAs, which affect the translation of AHR as well as “normal” target genes. This can fine-tune the magnitude and duration of the canonical AHR signaling pathway.^67,68^

### Noncanonical AHR pathways

Depending on additional context parameters, such as species, tissue and cell type, developmental stage, and micromilieu (e.g., healthy vs. inflamed vs. malignant conditions), the ligand-specific modulation of AHR may also drive several noncanonical functions of AHR. The latter often influences AHR-mediated regulation of immune responses, inflammatory processes, and other patho-/physiological functions.^27^ Only deeper mechanistic insight into the various noncanonical pathways and functions of AHR will enable us to harness its full therapeutic potential. As illustrated in Fig. 2, several modes of interaction exist by which AHR may crosstalk with other transcription factors and signaling molecules. These include but may be not limited to (i) transrepression, i.e., competition for the dimerization partner ARNT or transcriptional coactivators with other transcription factors; (ii) synergy on the basis of the accessibility of regulatory gene sequences; (iii) context-dependent interaction with alternative binding partners; (iv) induction of the proteasomal degradation of client proteins; and (v) stimulation of cellular signal transduction pathways by components of the cytosolic AHR multiprotein complex.

#### Transrepression

ARNT is an obvious candidate protein whose expression level and availability might affect the transcriptional activity of AHR.^69^ As shown in Table 1, ARNT serves as a heterodimerization partner for a variety of bHLH/PAS proteins, and accordingly, when one of those proteins is active, it may compete with AHR for ARNT binding and affect the AHR response. This is the case when the AHR repressor (AHRR) is expressed to inhibit the transcriptional activity of AHR by competing with AHR to form a heterodimer with ARNT.^70^ The AHRR-ARNT complex also binds to XRE, but instead of inducing transcription, it recruits transcriptional corepressors that attenuate gene expression.^71^ However, AHRR may not fully explain the complexity of AHR actions, and the extent to which AHRR regulates AHR signaling in all cell types and contexts remains to be fully characterized.

**Table 1:** Noncanonical interaction partners of AHR and ARNT, which can influence signaling outcomes

Abbreviation	Name	Direct binding/ligand dependency	Main function(s)	Reference
**AHR**				
ATP5α1	ATP synthase subunit	Interacts in absence of ligand binding (tandem affinity purification). Sequestration to mitochondria	Oxidative phosphorylation	^504^
Cul4B	Cullin 4B	Ligand dependent, direct binding in complex	E3 ubiquitin ligase involved in embryonic development, cell-cycle regulation, chromatin remodeling	^105^
ERα	Estrogen receptor α	Liganded AHR/ARNT complex associates with unliganded ER	Regulation of reproductive system function, bone homeostasis, vasodilation	^103^
KLF6	Krueppel-like factor 6	Heterodimer with AHR, in vivo chromatin immunoprecipitation, DNA binding studies, to nonconsensus XRE	Zinc finger transcription factor involved in controlling cell proliferation, differentiation and apoptosis	^505^
RB	Retinoblastoma protein	Direct binding RB and AHR, liganded AHR preferred	Cell-cycle control, G_1_-S transition	^506^
RelA/p65	NF-κB subunit	Physical interaction, coimmunoprecipitation assays, ligand-dependent	Development of the immune system, regulation of immune response to viral infection, inflammation, cell survival	^97^
RelB	NF-κB subunit	Physical interaction, results in different promoter-region requirements	Development of the immune system, regulation of immune response to viral infection, inflammation, cell survival	^93^
STAT1/STAT5	Signal transducer and activator of transcription 1 or 5	Physical binding, immunoprecipitation, Western Blotting. Ligand dependency unclear	Regulation of interferon response to viral infection, cell survival	^99^
STING	Stimulator of interferon genes	Physical interaction of the STING cyclic dinucleotide binding domain of STING1 with AHR	Sensing of cytosolic DNA, regulation of type 1 interferon response to bacterial or viral infection	^507^
**ARNT**				
AHRR	AHR repressor		Negative feedback regulator of AHR/ARNT signaling	^70^
HIF-1α	Hypoxia-inducible factor 1α		Oxygen sensing, adaptation to hypoxic conditions, regulation of glucose metabolism, angiogenesis	^508^
HIF-2α	Hypoxia-inducible factor 2α		Oxygen sensing, adaptation to hypoxic conditions, regulation of glucose metabolism, angiogenesis	^509^
HIF-3α	Hypoxia-inducible factor 3α		Oxygen sensing, adaptation to hypoxic conditions, regulation of glucose metabolism, angiogenesis	^510^
NPAS1	Neuronal PAS protein 1		Neuronal development and function	^126^
NPAS3	Neuronal PAS protein 3		Brain development and function	^126^
NPAS4	Neuronal PAS protein 4		Regulation of synaptic plasticity, circadian rhythm	^126^
RelB	NF-κB subunit		Development of the immune system, regulation of immune response to viral infection, inflammation, cell survival	^94^
SIM1	Single-minded protein 1		Brain development, neuroendocrine regulation, energy homeostasis	^511^
SIM2	Single-minded protein 2		Brain development and function	^511^

Another bHLH/PAS protein that is thought to inhibit AHR-regulated gene expression *via* competition for the common binding partner ARNT^69^ is hypoxia-inducible factor-1α (HIF-1α). In fact, several studies have shown that basal and inducible AHR activity is repressed under hypoxic conditions,^72,73^ and mechanistic studies on rodent hepatoma cell lines revealed ARNT as the limiting factor.^73,74^ However, in subsequent studies on human cell lines^75^ and murine HIF-1α-null hepatocytes,^76^ the competition of both transcription factors for ARNT could not be confirmed. Although these studies did not rule out the involvement of HIF-2α or HIF-3α, it is more likely that competition for the p300/CREB-binding protein (CBP) is causally involved. This transcriptional coactivator has histone acetylase activity,^77^ and its recruitment is essential for the transcriptional activity of both AHR-ARNT^78,79^ and the HIF-1α-ARNT complex.^80^ Competition for p300/CBP seems to be responsible for the mutual interaction of various transcription factors,^81^ including HIF-1α and p53, during hypoxia^82^ and AHR and NF-κB in hepatoma cells coexposed to TCDD and tumor necrosis factor-α (TNF) or lipopolysaccharide (LPS).^83^ In human keratinocytes, epidermal growth factor (EGF)-triggered activation of the EGF receptor (EGFR) and downstream signal transduction was reported to inhibit CYP1A1 transcription by preventing the recruitment of p300/CBP, but not the AHR-ARNT complex, to the *CYP1A1* gene promoter.^84^ This study did not identify the responsible transcription factor, but it might be AP-1, which is activated by EGFR signal transduction and requires p300/CBP for its transcriptional activity.^77^ Hence, competition for p300/CBP may be a central mechanism for the mutual interaction of the AHR-ARNT complex with HIF-1α and other transcription factors.

#### Synergy based on promoter accessibility

The opening of the local chromatin structure, for example, by histone acetylases, is also relevant with respect to the interaction of transcription factors, whose binding motifs are in close proximity in a given gene promoter/enhancer. Support for this idea is provided by studies showing synergistic induction of interleukin 6 (IL-6) in human breast cancer cell lines cotreated with IL-1β and the AHR agonists TCDD and kynurenic acid.^85,86^ Other mechanistic studies revealed that ligand-activated AHR binds to a nonconsensus XRE in the IL-6 promoter, leading to an opening of the local chromatin structure, which enhances the accessibility of the IL-1β-induced NF-κB complex.^87^ Similar synergistic induction has been observed for the proinflammatory chemokine (C-C motif) ligand 20 (CCL20) in AHR agonist- and LPS-treated macrophages^88^ as well as for NAD(P)H: quinone oxidoreductase, which harbors antioxidant-responsive elements (AREs) adjacent to XREs in its promoter sequence.^89^ Specifically, treatment of hepatoma cells with the bioactive nitrile crambene and acid condensation products of indole-3-carbinol (I3C) resulted in coactivation of the ARE and XRE motifs via the binding of nuclear factor erythroid 2-related factor 2 and AHR, respectively.^90^

#### Alternative binding partner

Depending on the cellular context and exposure situation, AHR may also interact with proteins other than ARNT (Table 1). For example, treatment of U937 macrophages with TCDD has been shown to induce the expression of IL-8 in a dose-dependent manner. Subsequent mechanistic studies revealed that this upregulation of IL-8 was mediated by AHR and protein kinase A (PKA) and did not require ARNT.^91^ Previously, treatment of hepatoma cells with the PKA activators cAMP and forskolin was reported to induce the translocation of AHR into the nucleus but not its heterodimerization with ARNT.^92^ The potential alternative dimerization partner of AHR remained unknown until the identification of a RelB/AHR binding element (RelBAHRE) in close proximity to the TATA box of the IL-8 gene, to which a complex consisting of AHR and the NF-κB subunit RelB binds to induce transcription.^91^ Functional RelBAHRE motifs were subsequently also identified in the regulatory sequences of other chemokines.^93^ Interestingly, ARNT has been shown to control CD30-triggered NF-κB activity in lymphoma cells by physically interacting with RelB at the promoter of NF-κB-responsive genes.^94^ As summarized recently in more detail,^27^ AHR also interacts with the NF-κB subunit RelA. For example, LPS enhances the ligand-induced AHR by inducing the binding of the RelA/p50 heterodimer to the promoter of AHR, thereby increasing its expression.^95^ A physical interaction between AHR and RelA has also been observed,^96,97^ for example, in breast cancer cells, in which AHR and RelA in complex transactivate the c-Myc promoter by binding to an NF-kB responsive element.^96^ As with NF-κB, interactions between AHR and members of the signal transducer and activator of transcription (STAT) protein family have been described at several levels.^27^ In particular, the direct protein interaction between AHR and STAT1 seems to be of functional relevance for diverse immune cell populations. In LPS-treated peritoneal macrophages, a tripartite complex consisting of AHR, STAT1, and the NF-κB subunit p50 was shown to occupy the IL-6 promoter and dampen its transactivation. Accordingly, both AHR and STAT1 deficiency were found to increase the LPS-induced expression of IL-6.^98^ In T cells, AHR sometimes facilitates polarization toward proinflammatory Th17 cells by forming a complex with STAT1, a negative regulator of Th17 development, thereby inhibiting its phosphorylation.^99^ In contrast, the same mechanism was reported to impair Th17 differentiation and promote Tregs in a model of idiopathic pneumonia syndrome.^100^ In this model, AHR represses the transcription of the AP-1 subunit JUND by preventing the binding of STAT1 to the respective gene promoter, resulting in reduced expression of IL-6 and associated polarization of CD4^+^ T cells toward the Th17 cell promoter.^100^ This discrepancy in describing AHR as an inducer of Th17 cells versus an inducer of Tregs has been previously discussed and may be due to different AHR ligands and/or applied doses.^27^ Importantly, AHR activation modulates T-cell differentiation into other subtypes and does not always affect CD4^+^ T-cell differentiation into Th17 cells,^101,102^ suggesting that AHR signaling works in concert with other signals to cue or guide T-cell differentiation and immune function.

#### Proteasomal degradation of client proteins

Several studies have reported that the activation of AHR by dioxin-like chemicals modulates or even disrupts endocrine circuits, particularly sex steroid hormone signaling.^103,104^ For example, TCDD treatment induces the proteasomal degradation of estrogen receptor-α (ERα). In 2007, AHR was identified as an integral part of an E3 ubiquitin ligase complex that transfers ubiquitin to client proteins and thereby targets them to the proteasome.^105^ This complex consists of AHR, the E3 ubiquitin ligase Cullin 4B (CUL4B), damaged-DNA binding protein 1, and several other proteins. Upon ligand-driven activation, AHR initiates the assembly of the so-called CUL4B^AHR^ complex and serves as a substrate-specific adapter protein, resulting in the proteolysis of ERα, ERβ, and the androgen receptor.^105^ A mechanistic follow-up study revealed a critical role for ARNT in this process.^106^ When ARNT is available, AHR acts as a ligand-activated transcription factor, resulting in the expression of target genes. When ARNT is not available, e.g., when it is occupied by other proteins, such as the AHRR, AHR seems to switch its functions and induce CUL4B^AHR^ complex assembly and client protein degradation. Moreover, other signaling proteins, including β-catenin, peroxisome proliferator-activated receptor-γ, and eukaryotic elongation factor-2 kinase, have been identified as substrates of the CUL4B^AHR^ complex.^107–109^ For example, PPARγ protein expression is decreased following AHR activation by FICZ in human THP-1 macrophages.^110^

#### Stimulation of signal transduction pathways

The activation of AHR leads to changes in protein phosphorylation pathways, such as those affecting mitogen-activated protein kinase (MAPK) and PI3K/AKT signaling.^27,111^ A key event for this seems to be the AHR ligand-triggered release of the soluble tyrosine kinase c-Src from the cytosolic AHR multiprotein complex.^64,65^ Active c-Src may directly activate EGFR in the plasma membrane by phosphorylating its intracellular domain at tyrosine residue 845.^112^ In addition, it can sequentially stimulate protein kinase C (PKC)^113^ and sheddases, such as ADAM17,^114^ resulting in the ectodomain shedding of cell surface-bound ligands of EGFR, such as amphiregulin (AREG) and transforming growth factor-α.^115^ These polypeptide growth factors activate EGFR in an autocrine or paracrine manner by binding to its extracellular domain and thereby induce downstream signal transduction pathways, such as the MEK–ERK axis, to alter cellular functions. In addition, c-Src was found to activate focal adhesion kinase, which may promote cell migration by fostering the clustering of integrins.^116^

## Structural basis of AHR function

**Fig. 3:**
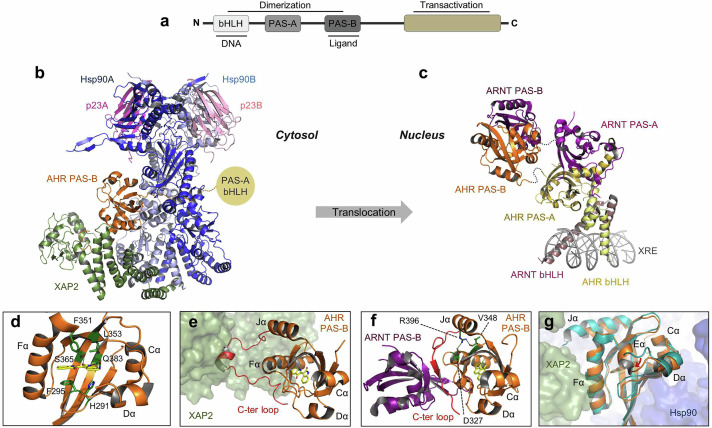
AHR structure. **a** Domain organization of members of the bHLH/PAS protein family. **b** Cryo-EM structure of the mouse AHR-Hsp90-XAP2-p23 cytosolic complex (Protein Data Bank, PDB ID: 8H77). All the components of the complex are labeled and displayed with a color code that is conserved throughout the figure. The high dynamics of the bHLH/PAS-A region hindered its visualization in the cryo-EM map, preventing the construction of the corresponding molecular model. **c** Crystal structure of the DNA-bound porcine AHR-ARNT heterodimer in complex with benzo[a]pyrene (PDB ID: 8XS8). The locations of the xenobiotic-response element (XRE) and bHLH, PAS-A, and PAS-B domains of AHR (yellow to orange) and ARNT (pink to purple) are labeled. The dotted lines between the PAS-A and PAS-B domains indicate invisible linkers. **d** Close-up view of the human AHR PAS-B ligand binding pocket bound to indirubin (PDB ID: 7ZUB). Key amino acids in contact with the ligand are labeled and shown as green sticks. Indirubin is shown as yellow sticks. The key helices are labeled. **e** Close-up view of the interaction of AHR PAS-B (orange) with the cochaperone XAP2 (green molecular surface) in the indirubin-bound human AHR-Hsp90-XAP2 complex (PDB ID: 7ZUB). The C-terminal loop (C-ter loop) of AHR PAS-B is colored red. Indirubin is shown as yellow sticks. **f** Overall structure of the PAS-B domains of the porcine AHR-ARNT heterodimer (PDB ID: 8XS8). The C-terminal loop of AHR PAS-B is colored red. Benzo[a]pyrene is shown as yellow sticks. Residues of the interaction network are labeled and displayed as green sticks. The dotted lines indicate hydrogen and electrostatic bonds. AHR PAS-B is in the same orientation as in (**e**) to facilitate comparison. **g** Superimposition of human benzo[a]pyrene-bound (PDB ID: 8QMO, colored orange) and unliganded mouse (PDB ID: 8H77, colored teal) AHR PAS-B structures showing the different paths of the Dα-Eα loop in the two structures (highlighted by a red arrow). Benzo[a]pyrene is shown as light orange sticks

The AHR belongs to the bHLH/PAS protein family, which contains sixteen members divided into two classes. Class I includes the hypoxia-inducible factors HIF-1α, 2α, and 3α; neuronal PAS proteins (NPAS1-4); single-minded proteins (SIM1, 2); the circadian regulator CLOCK; AHRR and AHR. These proteins form exclusive heterodimers with a class II member, the brain and muscle ARNT-like proteins (BMAL1, 2) or ARNT (also known as HIF-1β) and ARNT2.^117–119^ All these proteins share a common organization with essentially three functional domains (Fig. 3a). The N-terminal bHLH domain is involved in DNA binding and dimerization. The PAS domain exists as a tandem of two subdomains (A and B) encompassing ~50 amino acids each, with PAS-A involved in dimerization and PAS-B serving as a ligand binding and dimerization domain. The C-terminal part of the protein harbors an intrinsically disordered transactivation domain (TAD).

### AHR–ARNT heterodimerization and binding to DNA

To act as a transcription factor within the canonical signaling pathway, AHR must form a heterodimeric complex with ARNT. While the bHLH domains of both proteins are responsible for DNA binding, all three N-terminal domains, bHLH, PAS-A, and PAS-B, have been shown to contribute to the heterodimer interface.^120–122^ Several crystal structures of AHR-ARNT heterodimers, both single-domain and multidomain, have been reported, offering insights into the DNA binding mechanisms and dimerization of the complex. Early crystallographic studies of the isolated AHR PAS-A domain, combined with detailed mutational analysis, revealed the dimerization properties of the domain and identified hydrophobic surface residues essential for its heterodimerization with ARNT.^123^ At that time, efforts to produce soluble human AHR constructs containing the PAS-B domain were unsuccessful, leading to studies of DNA-bound AHR–ARNT heterodimers, which lack both protein PAS-B and TAD regions. Crystal structures obtained in two independent studies revealed that ARNT wraps around AHR, forming a highly intertwined, asymmetric architecture (Fig. 3c).^124,125^ These structures exhibit significant heterodimerization interfaces between the corresponding domains of each protein, featuring numerous hydrophobic interactions, as well as a few hydrogen bonds and salt bridges. The AHR-ARNT complex specifically recognizes the XRE core sequence 5’-NGCGTG-3’ (where N represents any type of nucleotide), with AHR binding to the 5’-NGC half site and ARNT recognizing the 3’-GTG half site. The bHLH domains of both proteins create a “tweezer-like” structure that interacts with the major grooves of the XRE. Residues in these domains form direct hydrogen bonds and salt bridges with the DNA bases and phosphate groups, ensuring precise recognition and tight binding to the target XRE sequence.

More recently, screening of AHR from various species resulted in the successful crystallization of the XRE-bound AHR-ARNT heterodimer, which consists of contiguous bHLH/PAS-A/PAS-B segments from porcine AHR and human ARNT. Six structures were determined in the presence of six different AHR ligands.^56^ In line with the agonist nature of the six ligands examined, all the structures obtained exhibited the same quaternary architecture. As expected, the observed quaternary architecture and DNA-binding mode of the bHLH/PAS-A domains closely resembled those reported previously and described above.^124,125^ Additionally, the structures revealed that the AHR-ARNT PAS-B heterodimeric unit is dynamic and spatially separated from the other protein domains (Fig. 3c). This contrasts with the previously determined HIF-2α-ARNT or NPAS1-ARNT structures, which display more compact shapes and extensive interaction surfaces between the PAS-A and PAS-B domains.^126–128^ This study also identified a long AHR PAS-B C-terminal loop unique to the AHR-ARNT heterodimer, which intercalates between the two PAS-B domains and forms numerous intra- and interdomain contacts (in red in Fig. 3f). While not strictly necessary for the formation of the full-length AHR-ARNT heterodimer, this loop may enhance heterodimerization of the PAS-B domains, thereby playing a significant role in AHR activation (see below “A model for ligand-dependent receptor activation” for further details). The specific features of AHR-ARNT heterodimers may be linked to the unique ligand-dependent activity of AHR within the bHLH/PAS protein family.^56^

### Mechanism of ligand binding to AHR

For three decades, structural studies of the ligand-binding PAS-B domain of AHR have been hindered by its highly aggregation-prone nature. Hence, numerous in silico studies have been carried out to predict the structure of the AHR PAS-B domain via homology modeling on the basis of the structures of closely related PAS domains, and its ligand-bound forms have been modeled through ligand molecular docking.^129^ While the structures of the PAS-B domains from other members of the bHLH/PAS-B family were determined in isolated or full protein contexts over 20 years ago,^118^ the first experimental images of AHR PAS-B were not revealed until 2022.

The first reported experimental structure of a mammalian PAS-B domain was that of human AHR in the form of a multiprotein cytosolic complex including chaperones and cochaperones.^54^ In this structure, AHR is bound to indirubin, a dietary-derived endogenous ligand produced from tryptophan by the intestinal microbiota. A subsequent structure published a few months later revealed the unliganded form of the mouse receptor, again within a cytosolic complex.^130^ Both structures were determined via cryogenic electron microscopy (cryo-EM), which revealed the coordinated action of the chaperone Hsp90 and cochaperones XAP2 and p23, which stabilize AHR in a stable, functional state (Fig. 3b). Reminiscent of the previously reported glucocorticoid receptor cytosolic complexes,^131–133^ the structures revealed a closed conformation Hsp90 dimer with AHR threaded through its lumen, leaving the two PAS domains at opposite faces of the chaperone dimer (Fig. 3b). In both structures, however, the bHLH and PAS-A domains could not be identified because of the highly dynamic nature of this region. XAP2 resides on the same side as PAS-B and extensively interacts with AHR and Hsp90. The AHR PAS-B domain interacts with the middle and C-terminal domains of Hsp90 subunits A and B, respectively, whereas on the N-terminal side, the linker between PAS-A and PAS-B weaves through the Hsp90 dimer, forming many contacts with both Hsp90 chains. The two p23 molecules are located on opposite sides of the N-terminal domain of the Hsp90 dimer.

In both structures, the ligand-binding PAS-B domain of AHR contains the canonical PAS fold comprising a five-stranded antiparallel β-sheet (Aβ, Bβ, Gβ, Hβ and Iβ) flanked by four consecutive α-helices (Cα, Dα, Eα and Fα) and two additional short α-helices (Jα and Kα) at the carboxy terminus.^54,130^ This arrangement generates a pocket of ~680 Å^3^ at the center of the domain, shaped as an elongated channel that runs perpendicularly between helices Fα and Cα (Fig. 3d). Like most AHR ligands, indirubin has a planar, aromatic structure. It occupies approximately half the volume of the binding pocket, with an anchoring point near the Fα helix. The binding site primarily consists of aliphatic and aromatic amino acids, along with a few polar residues, particularly S365 and Q383, which form hydrogen bonds with indirubin. Additional interactions with the ligand are predominantly van der Waals and hydrophobic, with several π- and CH/π-interactions occurring on either side of the ligand plane (e.g., H291, F295, F351, L353). This region of the pocket has been termed the “primary binding site” owing to its distinct composition and spatial arrangement, which govern the specificity and binding affinity of ligands to AHR. In contrast, the “secondary binding site”, located near the Cα helix and vacant in the indirubin-bound structure, could allow the receptor to accommodate larger compounds and likely contributes, in part, to the ligand-binding promiscuity of AHR. These conclusions were later confirmed by additional structures obtained with different ligands, either in the context of the cytosolic^55^ or AHR-ARNT^56^ complexes.

### A model for ligand-dependent AHR activation

To gain a deeper understanding of how ligand binding may influence AHR activity, several studies have been conducted to investigate the conformational changes in the AHR PAS-B domain across different ligation and activation states on the basis of various reported structures. While the overall fold of the PAS-B domain remains largely unchanged, structural superimposition revealed two loop movements that could play crucial roles in the regulation of AHR. First, a comparison of the ligand-free^130^ and ligand-bound^54,55^ PAS-B structures in the cytosolic complexes revealed that the loop connecting the Dα and Eα helices (Dα-Eα loop) undergoes a significant rearrangement upon ligand binding (Fig. 3g), resulting in some of its residues being in close contact with the ligand. Furthermore, this ligand-bound Dα-Eα loop conformation was also observed in both the liganded AHR-ARNT structures^56^ and the unliganded PAS-B structure of *Drosophila melanogaster* AHR,^134^ which lacks a known ligand and functions as a constitutively active receptor, similar to other invertebrate AHRs.^135,136^ This suggested the existence of a specific Dα-Eα loop conformation in AHR PAS-B active forms (e.g., ligand-bound). Notably, molecular dynamics simulations have previously shown that the region extending from the Cα helix to the Dα-Eα loop displays high flexibility, which is diminished upon ligand binding.^54,137^ Collectively, these findings indicate that this malleable region of PAS-B may play important roles in allowing compounds to reach the binding pocket and enabling the AHR to accommodate ligands of varying sizes, thus contributing to the promiscuity of the receptor. Finally, structures of ligand-bound AHR–ARNT complexes revealed that the Dα–Eα loop is an integral part of the dimerization interface with ARNT.^56^ Its stabilization upon agonist binding is therefore very likely to play an active role in the mechanism of AHR activation.

Second, a large rearrangement of the loop connecting PAS-B and the TAD (referred to as the C-terminal loop) was observed when comparing AHR PAS-B structures in the ARNT-bound and chaperone-bound states.^54–56^ In the AHR-ARNT heterodimer, this C-terminal loop is tightly associated with the AHR PAS-B core and appears to be a crucial component of the dimerization surface of this domain (Fig. 3f). In contrast, in the cytosolic complex, the C-terminal loop is dissociated from AHR PAS-B and instead interacts with the cochaperone XAP2 (Fig. 3e). Quite surprisingly, deleting this loop has a minimal effect on overall AHR-ARNT dimerization. Nevertheless, it significantly impairs AHR transcriptional activity.^56^ These findings suggest that the integrity of the PAS-B heterodimeric unit is necessary for AHR transactivation but is not essential for heterodimerization and XRE binding.^56,57^ Additional structural analysis revealed that the ligand-bound state of AHR PAS-B results in an interaction network that does not form in the absence of a ligand (Fig. 3f). This network involves amino acids of the ligand-binding pocket (V348 and F349, porcine numbering), D327 from the Fα helix, and R396 located at the junction of Jα and the C-terminal loop.^56^ On the basis of these observations, an allosteric mechanism linking ligand binding to conformational changes and receptor activation has been proposed. In this model, the presence of the ligand is sensed by the ligand binding pocket residues V348 and F349, and a signal is transmitted to the Jα helix through the newly formed interaction network, triggering the subsequent relocalization of the C-terminal loop. This, in turn, facilitates heterodimerization with ARNT and the dissociation of Hsp90 and XAP2 from the AHR-ARNT complex. Importantly, these large conformational changes and complex rearrangements, including the formation of a transient quaternary Hsp90-AHR-ARNT-XAP2 complex,^56,57^ are likely driven by ATP hydrolysis, which opens the Hsp90 dimer and relieves the structural constraints of the closed conformation.

In addition to the capacity of AHR to bind small regulatory molecules at the orthosteric site of PAS-B (e.g., indirubin or B[a]P), recent studies have revealed that carvones, a class of monoterpenoids, bind to the bHLH/PAS-A region and function as noncompetitive allosteric antagonists by blocking the heterodimerization of AHR with ARNT.^138^ Collectively, these findings may prove very useful for the development of new AHR modulators, either activators or inhibitors, on the basis of their capacity to influence the relocalization of the PAS-B C-terminal loop, heterodimerization with ARNT and DNA binding.

## AHR natural ligands and physiological context

AHR is a member of the bHLH/PAS protein family that is modulated through binding small molecules. After the discovery of this receptor in 1976, studies focused on the identity of high-affinity xenobiotics that are planar hydrophobic and composed of 3-6 aromatic rings.^3,139^ Ligand identification and characterization initially focused on halogenated dioxins and dibenzofurans, polycyclic aromatic hydrocarbons (e.g., benzo[a]pyrene, B[a]P) and indolo[3,2-b]carbazole (ICZ).^11^ Many of these ligands are also substrates for CYP1A1 and can lead to hydroxylation, which markedly reduces the AHR activation potential. An illustration of this concept has been established for ICZ upon mono- and dihydroxylation.^140^ Thus, CYP1A1 metabolism establishes an AHR negative feedback loop.^141^ In vivo evidence of intestinal feedback control was explored through the use of mice that constitutively express intestinal CYP1A1, leading to a dramatic reduction in diet-mediated activation of the AHR and a reduced immune response to infection.^142^ These findings support the concept that barrier tissue expression of relatively high levels of AHR and basal CYP1A1 expression are capable of clearing polycyclic aromatic compounds, thus attenuating systemic ligand distribution.

The AHR has been shown to be involved in a myriad of physiological responses, such as embryonic development and immune and epithelial cell differentiation. These observations have led to a search for physiologically relevant ligands. AHR is clearly activated or repressed by structurally diverse ligands (Table 2). However, distinguishing between compounds that are ligands that have been experimentally shown to bind to the ligand binding pocket and in vivo AHR activators is important. The first experiment to identify an agonist typically involves adding it to a cell culture system and determining whether *CYP1A1* is induced. While robust induction would likely suggest that it is a direct ligand, there are other possibilities. For example, a compound could block the metabolism of metabolically liable AHR ligands present in culture. Another issue that needs to be considered is that the AHR activation potential can be species dependent, and assessment of the activation potential in one species does not necessarily translate to other species, a point that is highlighted in Table 2.

**Table 2:** Endogenous modulators that contribute to the physiological activity of AHR

Origin^a^	Name	AHR Activity^b^	Reference
Host	L-kynurenine (L-Kyn)	Induces AHR activity in physiological range (µM). Product of TDO/IDO activity. Major product of endogenous tryptophan metabolism. Considered immunosuppressive.	^45,380^
Host	Kynurenine-CKA	Induces AHR activity (µM). Formed from spontaneous deamination of kynurenine. Rapid GSH/NAC conjugation.	^512^
Host	D-kynurenine	Induces AHR activity (µM). Physiological relevance unclear. DAOO facilitates conversion of D-Kyn to nonchiral kynurenic acid.	^513,514^
Host	Kynurenic acid (KA)	Induces AHR activity in physiological range (nM-µM). Greater affinity for human AHR than rodent. Downstream product of L-Kyn metabolism and DAOO D-Kyn deamination. Product of I3Pyr and ROS.	^85^
Host	Cinnabarinic acid	Downstream product of kynurenine metabolism. Product of 3-hydroxyanthrinilate condensation. Induces AHR activity (µM).	^515^
Host	Xanthurenic acid	Downstream product of kynurenine metabolism. Induces AHR activity (µM).	^85^
Host–microbe-spontaneous	6-formylindolo[3,2*b*]carbazole (FICZ)	Potent (pM) induction. Spontaneous tryptophan photooxidation or oxidative conditions product in vitro. Endogenous production predicted from detection of urinary sulfate conjugates. Product of yeast tryptophan metabolism. Detectable in isolates derived from seborrheic skin dermatitis. Potent CYP1 substrate. Considered most potent tryptophan derived activator.	^44,158,181,516,517^
Host–microbe-spontaneous	Indole-3-pyruvate (I3Pyr)	Induces AHR activity (µM). Product of tryptophan deamination. Unstable, generates multiple products including AHR activators.	^167,170,179,518^
Phytochemical-dietary-host–microbe-spontaneous	Indole-3-acetic acid (I3A)	Induces AHR activity in physiological range (µM). Abundant phytochemical. Although considered of microbial origin, detectable in germ-free mice.	^164,518–520^
Host–microbe	Indole-3-lactic acid (I3L)	Induces AHR activity (µM). Product of I3Pyr through host/microbial lactate dehydrogenase activity. Abundant in lactating mothers through activity of Lactobacilli/Bifidobacter. Although considered of microbial origin, detectable in germ free-free mice.	^164,518^
Microbial	Indole-3-acetamide	Induces AHR activity (µM). Product of microbial tryptophan metabolism.	^518^
Host–microbe	Indole-3-acetaldehyde	Induces AHR activity (µM). Product of microbial tryptophan metabolism.	^518^
Host–microbe	Indole-3-aldehyde	Induces AHR activity (µM). Although considered of microbial origin, detectable in germ-free mice.	^164,306^
Microbial	Indole	Induces AHR activity in physiological range (µM-mM) (fecal). Product of microbial tryptophan metabolism.	^307,518^
Microbial	Malassezin	Induces AHR activity (nM). Product of yeast tryptophan metabolism. Detectable in isolates derived from seborrheic skin dermatitis.	^155–159,521^
Microbial	Pityriazepin	Induces AHR activity (nM). Product of yeast tryptophan metabolism. Detectable in isolates derived from seborrheic skin dermatitis.	^159^
Microbial	Pyocyanin	Induces AHR activity (mM). Microbial origin, detectable in *P.aeruginosa* isolates. AHR sensing of Pyocyanin regulates host antimicrobial defense.	^259,263^
Microbial	1-hydroxyphenazine	Induces AHR activity (mM). Microbial origin, detectable in *P.aeruginosa* isolates. AHR sensing of 1-hydroxyphenazine regulates host antimicrobial defense.	^259,263^
Microbial	Phthiocol	Induces AHR activity (mM). Microbial origin, detectable in *M.tuberculosis* isolates. AHR sensing of Phthiocol regulates host antimicrobial defense.	^259,330^
Microbial	3-o-C12-L-HSL and HHQ quorum sensing molecules	Suppress 1-hydroxyphenazine mediated AHR induction (mM). Microbial origin, growth phase-specific quorum-sensing molecules. Detectable in *P.aeruginosa* positive cystic fibrosis aspirates (nM)	^263,522^
Phytochemical-dietary-microbial	Indirubin	Potent (nM) induction. Greater affinity for human AHR than rodent. Product of indigo naturalis microbial metabolism. Product of microbial tryptophan metabolism. Product of yeast tryptophan metabolism. Detectable in isolates derived from seborrheic skin dermatitis.	^157,159,523–525^
Microbial	2-oxindole	Induces AHR activity (µM). Product of microbial tryptophan metabolism.	^163,307^
Host–microbe	Indoxyl sulfate (I3S)	Induces AHR activity in physiological range (nM-µM). Greater affinity for human AHR than rodent. Uremic toxin produced by host metabolism of microbial indole.	^526^
Microbial	3-methyl indole	Induces AHR activity in physiological range (µM) (fecal). Product of microbial tryptophan metabolism. May influence colonic epithelial function through activation of AHR.	^307,527^
Microbial	Indole-3-propionic acid (I3P)	Induces AHR activity (µM). Product of microbial tryptophan metabolism. Potent antioxidant activity.	^164,307,518^
Phytochemical	Indole-3-carbinol (I3C)	Induces AHR activity (weak). Product of *brassica* consumption. Prone to acid condensation in stomach, generates large number of oligomeric products, some of which exhibit AHR activity.	^11^
Phytochemical-dietary-microbial-spontaneous	Indolo[3,2*b*]carbazole (ICZ)	Induces AHR activity in physiological range (nM-µM) upon feeding *brassica* genus of plants. Potent product of I3C acid condensation. Product of microbial tryptophan metabolism. Product of yeast tryptophan metabolism. Detectable in isolates derived from seborrheic skin dermatitis. Potent CYP1A1 substrate.	^11,156,157,159,528,529^
Phytochemical-dietary-host	Diindoylmethane (DIM)	Induces AHR activity (µM). Product of I3C dimerization.	^530,531^
Phytochemical-dietary-microbial-spontaneous	Tryptamine	Induces AHR activity in physiological range (nM-µM). Product of microbial tryptophan metabolism.	^343,518–520,532,533^
Microbial	2,8-dihydroxyquinoline	Induces AHR activity (nM-µM). Greater affinity for human AHR than rodent. Detectable in fecal samples.	^165^
Microbial	Tryptanthrins	Induce AHR activity in physiological range (nM-µM) upon microbial metabolism of indigo naturalis.	^523,534,535^
Host-dietary	7-ketocholesterol	Represses AHR activity within physiological range (µM). Associated with atherosclerotic plaques and fatty liver.	^536^
Microbial	Indolimines	Induce AHR activity (nM-µM). Proposed bacterial-derived genotoxins associated with colon cancer. Detectable in cecum of species-specific colonized mice.	^537^
Phytochemical & host–microbe	Flavonoids^c^	Induce and inhibit AHR activity, depending on structure. Abundant phytochemicals. CYP1 substrates, may activate AHR through inhibitory competitive metabolism of other AHR ligands that also manifest as CYP1 substrates, e.g., ICZ and FICZ. Undergo extensive host and microbial modifications.	^538–542^

Most of the above listed AHR modulators physiologically coexist, establishing a complex, metabolically dynamic, competitive metabolome that regulates endogenous AHR activity. Such complexity and redundancy of predominantly AHR agonists necessarily raises the question: how is AHR activity physiologically regulated in a cell-type or tissue-specific manner in the context of a largely persistent AHR metabolome? With a few notable exceptions, many of the endogenous AHR ligands exhibit weak affinity for AHR (relative to their anthropogenic counterparts, e.g., TCDD) and thus their concentrations are generally positioned at the pre or early exponential phase of a hypothetical dose response. As such, a relatively small change in concentration likely elicits a small but nonetheless physiologically relevant shift on the dose‒response curve. Tissue-specific AHR activity in the context of a fluctuating yet persistent AHR metabolome is likely a consequence of localized availability, e.g., microbial ligands contributing to AHR activity in the gastrointestinal tract but also tissue-specific differences in transport, influx/efflux and metabolism of individual AHR ligands. To date, the ADME characteristics of the individual constituents of the AHR metabolome is largely unknown despite the physiological significance and likely impact on role of AHR in disease pathology

^a^Origin indicates the dominant source

^b^AHR activity indicates agonist or antagonist-like activity with an indication of whether physiologically detected levels are likely to elicit AHR activity. AHR activity is presumed to be a consequence of direct ligand binding function. Although, with a few high-affinity exceptions (e.g., ICZ, FICZ and indirubin), technical limitations associated with nonspecific AHR binding of low-affinity modulators at the high concentrations required to displace a labeled high-affinity/specificity reference ligand e.g., TCDD, necessarily restrict the designation of bona fide ligand to many endogenous AHR modulators, especially when examined at physiologically detectable levels

^c^USDA Database for the Flavonoid Content of Selected Foods (https://www.ars.usda.gov/arsuserfiles/80400525/data/flav/flav_r03-1.pdf)

There are three major sources of natural AHR ligands; diet, microbes and the host are known to activate AHR. A wide range of foods and spices have shown AHR activation potential when cell culture reporter assays and extracts are used.^143–145^ However, much less is known about whether various spices and foods can significantly activate AHR at relevant amounts that humans may consume. The most notable exception to this is cruciferous vegetables, such as broccoli, which contain the phytochemical indole glucosinolates. The indole part is enzymatically released by myrosinases once the plants are chewed or cut. This leads to the generation of a number of metabolites, the most studied being indole-3-carbinol, which undergoes acid condensation in the stomach to form a number of oligomeric products, with ICZ exhibiting high affinity for AHR.^11^ Numerous studies have illustrated the protective effects of I3C or broccoli consumption from intestinal insults, and some of these established effects occur in an AHR-dependent manner.^52,146–150^ Another potentially important source of AHR ligands is brewed coffee.^151^ Roasted coffee extracts induced significant increases in the level of CYP1A1 expression in cultured cells, whereas the minimal activity was detected upon the addition of unroasted coffee extracts. This suggests that high-temperature roasting likely leads to pyrolysis products, such as heterocyclic amines and PAHs, which are known AHR ligands. Additionally, norharman, a known compound in coffee beans, was shown to activate AHR.^151,152^ Importantly, coffee extracts inhibited dextran sodium sulfate-mediated intestinal damage in an AHR-dependent manner, which is consistent with the findings of other studies showing the anti-inflammatory effects of AHR activation. Future studies should examine the AHR activation potential of other roasted or charcoal-broiled foods. Table 2 provides examples of naturally derived substances that have been shown to modulate AHR activity. Flavonoids are likely the most abundant plant phytochemicals that can modulate AHR activity. They have the general structure of two phenyl rings and a heterocyclic ring, and thousands of members can be divided into five distinct subgroups. Many members of the flavone and flavonol subgroups are AHR ligands and can be found at significant concentrations in many foods consumed by humans.^153,154^ These observations suggest that the AHR activation potential of a variety of foods, which could contain a complex mixture of AHR ligands of varying degrees of agonist and antagonist potency, coupled with unknown concentrations of individual ligands, absorption efficiency, potential for metabolism and in vivo half-lives, makes the overall modulation of AHR activity difficult to predict.

Microbes metabolize tryptophan into a myriad of AHR ligands/activators. Malassezia yeasts are widespread commensal microorganisms found on human skin that can produce malassezin, pityriazepin, ICZ, indole-3-carbaldehyde, indirubin, tryptanthrin, and FICZ, all of which are AHR agonists.^155–159^ Thus, the AHR acts as a sensor for the presence of yeast tryptophan metabolites and can respond by upregulating barrier protein products and increasing keratinocyte differentiation.^160,161^ Most cell types that reside in the skin express AHR, and the overall responses to AHR activation are likely context dependent, such as the type of insult, timing, and dose of ligand present.^162^ The microbiota in the GI tract is complex and varies as one moves down the tract. The intestinal tract, especially the large intestine, contains most of the microbes found in humans and is also the primary site of tryptophan metabolism. The most abundant tryptophan metabolites in human feces are also AHR agonists, including indole, 2-oxindole, indole-3 acetic acid, and 3-methylindole.^163^ The tryptophan metabolites present at lower concentrations in feces are tryptamine, indole-3-carboxaldehyde, xanthurenic acid, kynurenic acid, 2,8-dihydroxyquinoline, 3-methyl-2-oxindole, and 3-indoleacrylic acid. These metabolites can be found in mouse cecal contents and mouse serum.^163,164^ Interestingly, some of these ligands exhibit significantly greater activation potential for human AHR than for mouse AHR.^85,165^ Whether these serum tryptophan metabolites are derived from the gut microbiome or are generated by the host was recently explored in germ-free mice. The serum levels of indole-3-lactic acid, indole-3-carboxaldehyde, and indole-3-acetic acid are essentially the same in conventional and germ-free mice, suggesting that these serum metabolites are produced by the host.^164^ These metabolites appear to not be subject to a CYP1A1 feedback loop and thus may participate in basal AHR activity.^166^ The source of these metabolites is likely tryptophan deamination, resulting in the formation of indole-3-pyruvate (I3Pyr).

I3Pyr, the product of oxidative and enzymatic tryptophan deamination,^167^ has been demonstrated in vitro to elicit robust AHR agonist-like activity at low micromolar concentrations. Although tryptophan deamination to I3Pyr is viewed as an important component of plant and microbial tryptophan metabolism, its role in mammalian physiology is thought to be largely restricted to the intestinal microbiota. However, the discovery that mammalian aminotransferases exhibit a degree of promiscuity in terms of amino acid substrates and can catalyze the transamination of tryptophan to I3Pyr suggests that aminotransferases are involved in endogenous AHR activity in mammals.^168,169^ Evidence supporting aminotransferase AHR modulation was first demonstrated with aspartate aminotransferase (AST), which, in the presence of iron, induces AHR activity through the production of I3Pyr by amino transfer from tryptophan to alpha-ketoglutarate, yielding glutamate as a coproduct.^170^ This observation was further expanded with D-amino oxidase (DAOO), which, alone or in the presence of D-tryptophan, induces AHR activity through the production of I3Pyr, ammonia, and hydrogen peroxide.^171^ The physiological significance of DAOO-mediated AHR activity is unclear, given the scarcity of D-tryptophan in mammalian biology. Recent studies have identified an additional L-amino acid oxidase (LAOO) with the capacity to generate I3Pyr and subsequent AHR activity. The interleukin 4 (IL-4)-inducible target FIG1/IL4I1 (interleukin-four induced gene 1) was first characterized as an LAOO on the basis of its sequence homology with snake venom LOAA.^172^ The induction of IL4I1 by IL-4 suggests its involvement in T helper 2 (Th2)-mediated immunity and has been demonstrated to exert significant effects on autoimmune disease and cancer, particularly in the remodeling of the immune repertoire.^173–175^ Unlike aminotransferases and DAOO, IL4I1 is secreted and can promote tryptophan deamination at a distance, resembling tryptophan-2,3-dioxygenase (TDO)/IDO-mediated, SLC7A5/3A2 (solute carrier 7A5/3A2)-dependent transport of kynurenine under conditions of tryptophan depletion.^176,177^ Such spatial segregation is thus permissive for the dissemination of AHR activity beyond IL4I1-expressing cells.

Clearly, the transamination/deamination of tryptophan to I3Pyr by aminotransferases and DAOO and LAOO activity promote AHR activity and, given the ubiquitous and constitutive activity of aminotransferases, may contribute to both physiological and pathophysiological AHR ligand production. Despite the linkage between AHR activity and I3Pyr, its status as a direct AHR ligand has not yet been verified. Like dietary-derived indole-3-carbinol, I3Pyr is extremely chemically labile in aqueous and oxidative environments, generating numerous potential products.^167,170,171,173,178–181^ Many of the characterized I3Pyr-derived products have been shown to elicit AHR activity.^164^ Interestingly, products such as indole-3-acetate (and its downstream metabolites) and indole-3-lactate are reported as being exclusively microbiota dependent. Tryptophan trans-/deamination by multiple mammalian aminotransferases, LAOO and DAOO, would counter this assumption of microbial exclusivity.^173,174^ Indeed, studies have quantified significant levels of potentially I3Pyr-derived metabolites in the sera of germ-free mice.^164^

Given the dominance of TDO/IDO-mediated generation of kynurenine (and downstream metabolites), trans/deaminase-mediated I3Pyr (and downstream metabolite) production may represent a minor bystander effect toward the generation of physiological AHR activity. However, despite IDO1 having a greater catalytic rate, IL4I1 expression (and presumed activity) in tumors has greater predictive power regarding AHR transcriptional signatures (and tumor progression) than does TDO/IDO expression, indicating the physiological relevance of tryptophan deamination.^173,174^ The predictive nature of IL4I1 likely reflects the infiltration of CD11b^+^ myeloid cells into tumors, combined with the secreted nature of IL4I1 and the diffusion of I3Pyr.^175^ Furthermore, the production of I3Pyr is proposed to have clinical importance in circumventing therapeutic IDO inhibition, i.e., the switching of one AHR oncometabolite (e.g., kynurenine) for another (I3Pyr and its products).

Although not exclusive, it is evident that the majority of endogenous physiological AHR activation is ultimately a function of host/microbe tryptophan metabolism regardless of the species. As such, the AHR behaves as a tryptophan metabolite sensor, perhaps as a means of sensing microbial activity and responding by increasing epithelial barrier function and a heightened immune response. Consequently, it can be argued that endogenous tryptophan-related AHR physiology requires protection from external (e.g., diet and xenobiotic) activation, hence the capacity of AHR to maintain basal and inducible CYP1A1 levels. Furthermore, the homeostatic biological imperative for such a sensor function is impossible to ascribe but may be attributed to the fact that tryptophan, which is the most limiting essential amino acid, is thus an acutely sensitive indicator of physiological change, e.g., flux in dietary consumption or microbial dysbiosis. Nevertheless, a sensor (e.g., AHR) with the capacity to elicit physiological adaptation to a change in tryptophan metabolism contributes to organismal homeostasis and thus survival.

## AHR in development

As stated above, AHR is a ligand-activated transcription factor that was first recognized for its role in mediating the toxic effects of environmental pollutants. However, over the past two decades, many studies have revealed its critical functions in various aspects of developmental biology. This section explores the multifaceted roles of AHR in embryonic development, tissue differentiation, and organ system maturation, highlighting its importance beyond xenobiotic metabolism.

### Evolutionary conservation of AHR

The AHR pathway is highly conserved across species, from invertebrates to mammals, underscoring its fundamental importance in several biological processes.^182^ In invertebrates such as *Caenorhabditis elegans* and *Drosophila melanogaster*, AHR orthologs (AHR-1 and spineless, respectively) play crucial roles in neuronal development and cell fate determination.^183^ In *C. elegans*, AHR-1 regulates the fate of GABAergic motor neurons involved in head movement, controls axon branching and proper cell migration, and subsequently influences social feeding behaviors by regulating specific neuronal circuits.^184^ Spineless in *D. melanogaster* affects dendritic branching in sensory neurons and is crucial for the formation of sensory bristles and photoreceptor differentiation.^185^ Unlike mammalian AHR, these orthologs are believed to function independently of ligands, whereas some modulators have been identified^136^; the conservation of some developmental functions (e.g., dendrite and axon branching) across such diverse species suggests that these roles predate the AHR xenobiotic-sensing functions in vertebrates.^186^

In vertebrates, the AHR gene has undergone duplication events, resulting in multiple paralogs with potentially diverse functions.^187^ For example, fish possess multiple isoforms of AHR due to a fish-specific whole-genome duplication ~350 million years ago.^188^ This has resulted in at least two primary AHR isoforms, AHR1 and AHR2, which are further diversified into subtypes such as AHR1a, AHR1b, and AHR2 in some species, such as the zebrafish *Danio rerio*.^188,189^ These isoforms have undergone subfunctionalization, partitioning ancestral functions or developing new roles (neofunctionalization). For example, while AHR2 is the principal receptor mediating both toxicological responses to environmental contaminants and essential developmental processes in zebrafish, AHR1a and AHR1b play more tissue-specific roles; AHR1a is involved in gene regulation in organs such as the liver and brain, and AHR1b potentially influences eye development and function.^188^ Additional duplications in salmonids have led to even greater diversity, with species such as Atlantic salmon possessing multiple copies of both the AHR1 and AHR2 genes.^190^ Despite this diversification, core developmental roles have been maintained, particularly in the regulation of cell differentiation and organ development.

### AHR in embryonic and organ development

AHR expression and nuclear localization are detected early in embryogenesis. Its activation influences various aspects of embryonic development. One of the most striking observations is the involvement of the receptor in maintaining embryonic stem cell pluripotency. Under normal conditions, AHR is repressed in pluripotent cells, and its untimely activation leads to a loss of pluripotency, mitotic delays, and differentiation.^191^ Indeed, AHR negatively regulates key pluripotency factors such as OCT4, NANOG, and SOX2 by binding to repetitive genomic elements such as Alu, which triggers the production of noncoding RNAs that interfere with the expression of these genes.^192,193^ Additionally, AHR contributes to epigenetic modifications, such as DNA methylation, which further influence cell fate decisions.^191^ Studies have shown that AHR-null embryos overexpress pluripotency markers and exhibit delayed differentiation, highlighting the role of AHR in balancing self-renewal and differentiation processes during early development.^194^

One of the first organs studied in *Ahr*^-/-^ knockout mice was the liver. While different knockout models have been developed, one has shown that AHR genetic ablation reduces liver size and alters hepatic vascular architecture, indicating its importance in liver development. Moreover, bile duct formation was also altered.^19,195^

The hematopoietic system is another major target of AHR-mediated developmental regulation.^196^ For example, AHR inhibition has been shown to block the differentiation of mouse and human hematopoietic stem cells, thereby promoting their expansion.^197,198^ AHR also influences the development of various lymphoid cell types, including Th17 cells and Tregs, two critical subsets of CD4^+^ T cells, thus contributing to immune tolerance and inflammation regulation.^199^ AHR promotes Treg differentiation by increasing the expression of Foxp3, the master transcription factor for Tregs, through mechanisms involving endogenous and dietary ligands such as kynurenine and indole-3-carbinol.^200^ AHR also transactivates gut-specific Treg functions by facilitating their homing to the intestinal mucosa *via* the upregulation of markers such as GPR15, which are essential for maintaining gut immune tolerance.^201^ Conversely, AHR also supports Th17 cell differentiation by inducing the transcription factor RORγt. A ligand-dependent effect allows AHR to mediate proinflammatory responses through IL-17 and IL-22 production while simultaneously contributing to immune suppression *via* Tregs. Furthermore, ex vivo treatment of human cord blood hematopoietic stem cells with TCDD diminished B lymphopoiesis, in part by reducing the expression of transcription factors that control B-cell lineage commitment, such as PAX-5.^202^ The balance between these opposing roles is crucial for determining immune outcomes, as dysregulation of AHR signaling can lead to autoimmune diseases or chronic inflammation.^38,203,204^ AHR activation also affects the differentiation of innate lymphoid cells (ILCs), particularly ILC3s, which are critical for the homeostasis of the intestine,^205^ together with myeloid lineages and the differentiation of monocytes and Langerhans cells at an early precursor stage^206^ and ultimately the erythro-megakaryocytic axis.^207^ In conclusion, AHR functions as a “molecular rheostat” within hematopoietic progenitor cells, modulating lineage commitment and terminal hematopoietic phenotypes. Its diverse effects on HSCs and lymphoid and myeloid lineages make it a crucial factor in maintaining hematopoietic balance and immune system development.

During cardiovascular development, AHR contributes to the formation of the heart and major blood vessels.^208^ Moreover, one strain of AHR-null mouse displays patent ductus venosus, a condition in which the ductus venosus fails to close after birth, leading to liver problems.^209,210^ This phenotype highlights the role of AHR in proper vascular remodeling during development.

The functions of AHR in neurodevelopment have been extensively studied, particularly in *C. elegans*^211^ (see above). In vertebrates, AHR influences neuronal differentiation and migration in the developing brain.^212,213^ Studies in mice have shown that AHR is involved in the development of GABAergic neurons in the ventral telencephalon.^214^ In the hippocampus, AHR activation by tryptophan metabolites from the microbiome modulates adult neurogenesis.^215^ In addition, AHR also regulates other neural cell types, such as oligodendrocytes and Schwann cells. AHR is involved in both central and peripheral myelination, where its ablation results in thinner myelin sheaths surrounding axons, which is associated with locomotion defects or horizontal nystagmus. The identified mechanisms involve inflammatory responses and the regulated expression of myelin genes.^216,217^ The involvement of AHR in myelin gene regulation suggests that AHR could be a potential target for treating myelin-related disorders or nerve tumors.^218^

In addition to its role in embryonic development, AHR continues to play important roles in tissue homeostasis, repair, and regeneration in adult organisms.^219^ In the liver, AHR is involved in hepatic progenitor cell activation and differentiation during liver regeneration.^220^ This function has potential implications for the treatment of liver diseases and regenerative medicine approaches. In the skin, AHR activation promotes epidermal differentiation and barrier function.^221^ It also regulates the proliferation and differentiation of epidermal stem cells, contributing to wound healing processes.^222^ The regulation of these mechanisms, which involve cell migration, is reminiscent of metastatic pathophysiological processes.^61,223,224^

**Fig. 4:**
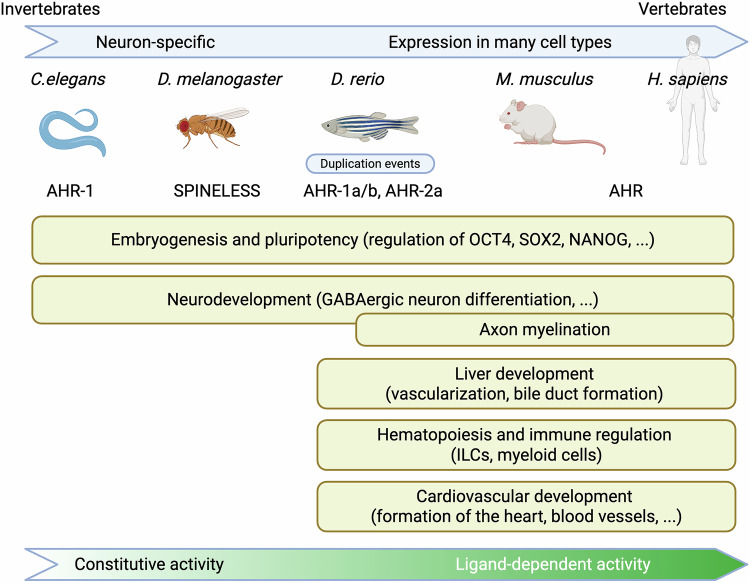
Evolutionary conservation and developmental roles of the AHR. This figure shows the evolution of the AHR from invertebrates to vertebrates. In invertebrates such as *C. elegans* (AHR-1) and *D. melanogaster* (spineless), AHR is mainly neuron specific. In vertebrates such as zebrafish (*D. rerio*), mice (*M. musculus*), and humans, AHR is expressed in many cell types, with several organisms, such as zebrafish, showing gene duplications (AHR-1a/b, AHR-2a). The multiple functions of AHR include the regulation of embryogenesis, neurodevelopment, liver and cardiovascular system formation, and immune cell differentiation. The receptor has evolved from constitutive activity in invertebrates to ligand-dependent signaling in vertebrates, reflecting its expanded role in development and environmental sensing. The figure was created via BioRender

In conclusion, AHR plays a pivotal role in development (Fig. 4) by engaging with key molecular pathways such as the Wnt/β-catenin, Notch, and transforming growth factor-β pathways, enabling it to influence cell fate decisions and differentiation in a context-dependent manner.^225^ At the transcriptional level, AHR governs the expression of genes critical for developmental processes and cancer progression.^226,227^ Given its broad regulatory functions, AHR is a crucial factor in cellular differentiation, organogenesis, and tissue homeostasis from early embryonic stages to adulthood. Future research should focus on revealing the precise molecular mechanisms underlying the interactions of AHR with developmental pathways, its role in human developmental disorders, and its potential applications in regenerative medicine and tissue engineering. Understanding the crosstalk between the developmental roles of AHR and its function in xenobiotic metabolism may provide new therapeutic opportunities. Expanding our knowledge of AHR in developmental biology could pave the way for novel interventions in developmental disorders and regenerative therapies.

## AHR in immunity and inflammation

The immune system is a complex network of organs, cells and soluble factors that collectively protect the body from infection and cancer and repair damage following injury. Immune cells are continuously produced and circulate throughout the body. In an orchestrated manner, immune cells are responsible for executing appropriate responses at the appropriate location and for the correct amount of time. Insufficient immune function increases susceptibility to infectious disease and cancer, whereas overly active or inappropriately targeted responses cause or exacerbate pathology *via* damage to healthy tissue. Hence, properly balanced immune responses are critical, and even subtle changes can have far-reaching consequences for communicable and chronic diseases.

There is abundant and persuasive evidence that the AHR modulates the immune system, with consequences ranging from subtle to overt. This evidence includes AHR-mediated impacts on hematopoiesis and infectious, autoimmune, allergic, and inflammatory responses.^228^ This evidence is derived primarily from studies of AHR agonism, but studies of absence and AHR antagonism provide additional support for AHR-mediated immune system effects.^53,228,229^ Notably, AHR is particularly intriguing because engaging in AHR differentially alters the frequency or function of a type of immune cell in different ways and can worsen *or* improve immunopathology.^38,39,101,230–236^ The specific consequences observed appear to be highly dependent on the organ, tissue, and cell type; immunological stimulus; timing relative to antigenic challenge; and nature of AHR engagement.^228^ This variability creates challenges in predicting whether and in what manner a molecule that binds to the AHR influences the immune system. However, what currently appears to be inconsistencies creates exciting opportunities to advance the field.

The initial discovery that AHR modifies the immune system was rooted in toxicology and the study of AHR binding to pollutants. Consequently, environmental contaminants, particularly TCDD, are among the most widely used AHR ligands. TCDD has many attractive properties as a tool to study AHR, including that it is an exclusive AHR agonist and that it is poorly metabolized. These features reduce confounding factors, such as secondary metabolites and the comodulation of other receptor-mediated pathways. TCDD binds to AHR with very high affinity and has a relatively long in vivo half-life; therefore, it does not need to be administered repeatedly. However, these properties pose challenges for the use of TCDD because even minute amounts elicit robust and durable AHR activation, which cannot be easily turned off. Despite these limitations, research using TCDD has revealed much about the impact of AHR on the immune system, including the finding that AHR activation can be immunosuppressive.^237,238^ There are now numerous examples of nonpollutant AHR ligands, such as tryptophan metabolites and derivatives, that cause immunomodulatory effects similar to those of TCDD.^101,102,231–234,239–241^ However, not all AHR ligands behave like TCDD does, and there are instances in which AHR ligands, such as FICZ, cause immunological consequences that are distinct from those of TCDD.^38,101,231^ The reasons for these similarities and differences are not fully understood, and only a handful of studies have directly compared the effects of multiple AHR ligands on multiple metrics of immune function.^38,101,102,242^

AHR is broadly expressed in mature immune cells, hematopoietic stem and progenitor cells, and nonhematopoietic cells throughout the body.^53,228^ This is an important concept because there is empirical evidence that AHR ligands have direct and indirect effects on immune cells. Evidence of lineage autonomous effects includes studies in which the in vitro addition of an AHR antagonist increased the proliferation of human hematopoietic stem and progenitor cells^197^ and that AHR ligands directly modulate some aspects of B-cell lineage development and differentiation.^202,243,244^ However, in other instances, AHR signaling influences immune responses indirectly. This includes indirect impacts of AHR ligands on aspects of lymphopoiesis,^245,246^ which also illustrates that AHR ligands can affect a lineage *via* a combination of direct and indirect effects. There is experimental evidence that AHR signaling in one cell type alters the response of another cell type. For example, in experimental autoimmune encephalomyelitis (a mouse model of multiple sclerosis), AHR activation with the high-affinity ligand ITE decreased lymph node cell proliferation *via* the induction of regulatory Tregs and tolerogenic dendritic cells (DCs).^247^ Similarly, AHR activation reduces clonal expansion and differentiation of CD8^+^ T cells into effector cytotoxic T lymphocytes during influenza A virus infection through an indirect mechanism that requires AHR signaling in DCs but not in CD8^+^ T cells.^248,249^ In other contexts, AHR activation indirectly affects lymphocytes by increasing the frequency of myeloid-derived suppressor cells.^250^ Furthermore, AHR signaling can influence immune cells *via* AHR-driven events in nonhematopoietic cells, including epithelial and endothelial cells.^49,251^ Therefore, when considering the mechanisms *by* which AHR signaling affects an immune response, it is vital to discern the contributions of direct and indirect mechanisms and recognize that both may be at play.

Evaluating the effects of AHR on the immune system in a holistic manner is crucial because even well-studied AHR ligands produce unexpected results.^252^ For example, TCDD exacerbated interstitial lung inflammation and increased morbidity and mortality in mice during acute infection with influenza A virus.^253,254^ However, the same dose of TCDD administered via the same route to the same mouse strain caused the opposite effect during respiratory infection with *Streptococcus pneumoniae*; that is, the mice survived better and had less pulmonary inflammation than did the infected controls.^230^ This dichotomy is unlikely due to the simple explanation of bacterial versus viral infection because, during ocular infection with a herpes simplex virus, neutrophilic inflammation is reduced in mice treated with TCDD.^255^ Additionally, in both viral and bacterial infections, pathogen-specific antibody responses were suppressed in mice given TCDD. These studies further illustrate that AHR activation with the same ligand can trigger similar and distinct immunomodulatory consequences and that these events can cooccur. While the mechanisms *by* which AHR causes these various consequences remain to be fully revealed, these examples emphasize that it is essential to examine multiple aspects of the immune response in more than one cell type.

This complexity likely explains some of the variability and suggests that AHR signaling influences how immune cells sense their local microenvironment to fine tune immune and inflammatory responses. In other words, AHR may influence the immune system in concert with other signals. Observations that support this idea include that AHR agonism, antagonism or absence are often insufficient to cause a discernable impact on the immune system without another stimulus. For example, in human population studies, there is limited or no association between exposure to AHR-binding pollutants and the steady-state frequency of circulating leukocytes^34,256^; however, the severity of infection is inversely related to exposure.^257,258^ Many in vivo studies have shown no discernable impact of AHR ligand treatment on the immune system in the absence of stimulating immune cells (e.g., by adding an antigen or mitogen). Further evidence comes from studies using mice that lack *Ahr* (i.e., global *Ahr*^*-/-*^ knockout mice). For many metrics, immune cellularity at a steady state is not different between *Ahr*^*-/-*^ knockout mice and wild-type mice. However, this is not always the case, which provides further evidence that context matters. Additionally, *Ahr*^*-/-*^ knockout mice are less able to fight infection with certain pathogens, such as *Listeria monocytogenes* and *Citrobacter rodentium*, but do not demonstrate any appreciable difference in host resistance to *Streptococcus pneumoniae* infection or immune challenge with nonpathogenic antigens.^230,259–262^

In fact, the AHR has been proposed as a sensor of bacterial quorum to sense the dynamics of infection and better orchestrate host defenses.^263^ AHR may also play a role in programming the immune system as it develops. Research has shown that early-life exposure to pollutants that bind AHR can have long-lasting effects on CD4^+^ and CD8^+^ T cells, which can be transferred to unexposed hosts.^264,265^ Consistent with the idea that AHR influences the immune system in concert with other signals, differences in CD4^+^ and CD8^+^ T cells are apparent only after immune challenge, and this effect is mediated, at least in part, through alterations in DNA methylation.^266,267^ However, for most AHR ligands, whether they are synthetic or naturally derived, their ability to influence the developmental programming of the immune system is largely underexplored.

While a multitude of AHR ligands have been identified, ranging from pollutants to pharmaceuticals to pathogenic and commensal microorganisms,^53,229,268–272^ AHR-driven immune modulation by many of these small molecules remains unexplored. For example, many AHR-binding chemicals have been identified via in vitro screening assays.^53,229,268^ However, the ability of many of these chemicals to modulate the immune system, especially in vivo, has yet to be rigorously examined. Additionally, for many of these compounds, information regarding their adsorption, distribution, metabolism and excretion is limited. Therefore, although there is significant evidence that AHR influences the immune system, many questions remain regarding how AHR (and various AHR ligands) modulates the development and function of the immune system. Given that hematopoiesis and immune responses are complex and tightly regulated processes that involve cell‒cell interactions, cellular maturation and differentiation, migration, proliferation and cell death, numerous processes can be influenced by AHR, which provides fertile ground for future research. Unraveling how AHR causes such a variety of consequences will involve integrating a range of approaches, such as spatiotemporal mapping of how AHR influences immune responses over time, across organs or tissues, and among different cells, including functionally distinct subpopulations as they change over the course of an immune response.

Overall, multiple studies support that AHR signaling influences the immune system in a highly context-dependent manner, as AHR agonism, antagonism or absence elicits cell type-, tissue compartment-, and disease-specific consequences for the immune system. This makes it challenging to make a single generalized statement about the impact of AHR signaling on the immune system. Nevertheless, this information suggests that AHR-binding chemicals may be useful for the prevention or treatment of immune and inflammatory diseases.

## AHR roles in barrier organs

By definition, barrier organs are the first line of defense against external challenges. The skin, lungs, and gut epitomize this role through specialized structures and cellular compositions that serve as critical interfaces between the body and the external environment, playing a central role in host defense. These organs are equipped with multilayered protective mechanisms, including physical barriers (e.g., epithelial cell layers), chemical defenses (e.g., antimicrobial peptides, mucus), and immune surveillance systems that detect and respond to different threats, including pathogens. Integral to their function is the resident microbiota, a diverse community of microorganisms that contributes to maintaining barrier integrity, modulating immune responses, and controlling potential pathogens.^273,274^ The dynamic interactions between barrier tissues, immune components, and the microbiota form a highly coordinated defense network that protects against infection and other pathological conditions and promotes immune homeostasis. Disruptions to this delicate balance, such as through injury, food contaminants, antibiotic use, or dysbiosis, can compromise host defense and contribute to various inflammatory, autoimmune, and infectious diseases.^273–275^

Barrier organs deploy a multifaceted immune response to protect against pathogens while maintaining tolerance to beneficial microbiota. These responses are orchestrated by an array of receptors, including pattern recognition receptors (PRRs), which can detect microbial- and danger-associated molecules and trigger immune responses. The microbiome plays a key role in this intricate network by interacting with these receptors and eliciting signaling pathways to reinforce barrier integrity and regulate host immunity, including the production of antimicrobial peptides. This dynamic interplay ensures that barrier organs can swiftly react to potential threats while maintaining the delicate equilibrium necessary for overall health.^275,276^

Among these PRRs is the AHR, which is strongly expressed in the skin, gut and lungs and, owing to its ability to sense a diverse range of ligands (Table 2), places it at the nexus of environmental, dietary, and microbial influences on host physiology, playing a pivotal role in regulating barrier organs and contributing to homeostasis and disease states.^46,53,63,252,259,277–279^ In the gut, skin, and respiratory tract, AHR signaling supports balanced interactions between host cells, the microbiota, and the environment, fostering tightly controlled cellular and tissue responses that are essential for barrier function.^46,53,277,280–282^ Remarkably, the interplay between AHR and the microbiome is bidirectional, whereby microbial metabolites (e.g., quorum-sensing molecules and indole derivatives, Table 2) can impact AHR, while AHR signaling in turn shapes the composition and function of the microbial community and its metabolome through the induction of metabolizing enzymes.^252,259,263,280,283^ Consequently, in barrier organs, this tightly regulated crosstalk plays a crucial role in shaping cellular, tissue, and organismal responses in diverse health and pathological conditions. Accordingly, dysregulated AHR activity can contribute to chronic inflammation, impaired barrier function, and even tumorigenesis.^46,53,63,252,259,277,279,280^ Owing to its central role in maintaining barrier homeostasis, AHR has emerged as a promising therapeutic target for a variety of conditions associated with barrier dysfunction, including autoimmune and inflammatory disorders and infection.^252,281,282,284–286^

As the body’s primary interface with the environment, the skin serves as a frontline defense against environmental stresses and pollutants (e.g., UV radiation), pathogens, and injury. AHR is widely expressed in skin cells, including melanocytes, keratinocytes, fibroblasts, and skin immune cells (e.g., Langerhans cells (LCs), γδ T cells, Th17 cells, ILCs, and DCs), although differences in constitutive or inducible levels of AHR in the diverse cell types that compose the skin layers may be observed.^53,277,287,288^ Similarly, the expression of AHR-regulated genes also differs across different cell types and/or proliferation and differentiation stages. For example, in addition to CYP1A1, whose levels increase during keratinocyte differentiation, AHRR levels also vary according to different cell types (e.g., high levels in LCs and fibroblasts).^161,288–290^ Therefore, different levels of CYP1A1 (important for the metabolism of diverse AHR ligands, including endogenous ligands) and AHRR also impact AHR signaling and its regulated effects.^46,142,279,291,292^ Multiple roles of AHR in the skin have been revealed (extensively reviewed in.^53,277,281,287,288,293,294^ These include the impact on keratinocyte differentiation and skin barrier integrity, whereby AHR deficiency (*Ahr*^-/-^) or AHR inhibition leads to significantly lower expression of keratinocyte differentiation markers such as loricrin, filaggrin, and hornerin.^51,161,277,288,295^ Conversely, the expression of those markers increases upon exposure to several AHR-activating conditions (e.g., FICZ, coal tar, tapinarof, lawsone).^59,288,295–297^ The AHR also regulates melanocyte proliferation and pigmentation. For example, AHR-regulated differences in pigmentation are observed upon UV exposure or exposure to chemical AHR agonists (e.g., bezanthrone, TCDD).^53,293,298,299^ A role for AHR in *vitiligo* (an autoimmune skin disease characterized by alterations in pigmentation) has also been shown.^300,301^ An impact on different skin inflammatory diseases (e.g., psoriasis, AD, *hydradenitis suppurativa*, and acne), the skin microbiome, and skin infections has also been documented.^53,277,281,288,293,294^ In psoriasis, a lack of AHR signaling is linked to exacerbated inflammatory conditions, including increased expression of proinflammatory cytokines (e.g., TNF, IL17a) and neutrophilia, as well as reduced keratinocyte differentiation, which can be reversed in the presence of AHR agonists, such as FICZ and tapinarof.^59,162,286,288,294,302^ Importantly, targeting AHR *via* tapinarof has been tested and recently approved for the treatment of psoriasis.^286^ The role of AHR in psoriasis is well established, and links between AHR modulation and AD pathogenesis and the deregulation of the AHR pathway in AD patients have been extensively reported.^161,281,288,293,294,303^ Curiously, coal tar (a product rich in AHR ligands) has been historically used as a topical therapy for AD, and it is now known that the activation of AHR (e.g., by coal tar) restores barrier integrity in different AD models and ameliorates disease severity, including by restoring epidermal differentiation (e.g., expression of filaggrin), sensing and impacting the skin microbiome (e.g., altering the prevalence of *Staphylococcus* and *Propionibacterium*, sensing *Malassezia* metabolites) and regulating the expression of antimicrobial peptides.^158,162,288,294,296,297,304^ In an in vitro and rat model of acne, microbiota-derived indoles were shown to attenuate inflammation and acne pathology *via* AHR.^305^ Furthermore, the crosstalk between AHR and the commensal microbiota has also been shown to impact resistance to skin bacterial infections, such as *Staphylococcus aureus*, in a tape stripping infection model.^51,281^ Overall, AHR plays multiple roles in the skin under both homeostatic and pathological conditions.

Like the skin, the GI tract is a large mucosal surface of the body that plays a major role in host protection against the environment and its threats (e.g., infection) while maintaining its key function of nutrient absorption, which is key to life. The GI tract is a complex “bioreactor” where the host and a vast array of microbial species coexist and interact and where a tightly regulated balance between both is of utmost importance to maintain body homeostasis and control different pathological conditions. It is therefore not surprising that, as the skin, multiple functions of AHR in the GI tract have been discovered.^46,63,278,280,282,284^ These mechanisms span key roles in the sensing of endogenous, microbial, and food-derived metabolites (e.g., indoles, Table 2), shaping the microbiome and metabolite availability (e.g., differential microbial composition and metabolite abundance between WT and *Ahr*^-/-^ mice), the generation and maintenance of different cell populations (e.g., intraepithelial lymphocytes and ILCs), tissue regeneration (e.g., AHR control of colonic epithelial differentiation), the regulation of inflammatory mediator expression (e.g., promoting the production of IL-22, a cytokine essential for epithelial regeneration and antimicrobial defense), and the regulation of gut motility (e.g., *Ahr*^-/-^ negatively impacts gut peristalsis with an impact on bacterial composition).^46,52,142,262,278,279,282–284,306–315^ The impact of AHR in GI diseases has also been shown, for example, in inflammatory diseases (e.g., IBD, such as Crohn’s disease and ulcerative colitis), microbial infections (e.g., *Citrobacter* and *Cryptosporidium* infections), and cancer (e.g., colorectal cancer).^46,149,252,262,278,282,316–320^ Importantly, for the skin, therapeutic targeting of AHR in the GI tract, for example, by diet or dietary supplementation with AHR ligands or microbes (e.g., indoles, probiotics), impacts the outcome of different diseases, from inflammatory to infectious diseases.^46,278,282,320–324^

The lungs are continuously exposed to AHR ligands that may arise from environmental pollutants (e.g., vehicle exhaust, cigarette smoke), microbes (e.g., bacteria, fungi), allergens, and other sources. Hence, maintaining lung homeostasis involves complex interactions among structural cells, immune components, and microbes, and the AHR has also emerged as a key modulator. Although less abundant than in the gut, the lung microbiota plays a significant role in shaping pulmonary immunity, where the AHR acts as a molecular sensor that connects microbial metabolic activity with host immune regulation. Recent studies have indicated that in addition to environmental pollutants, microbiota-derived, pathogenic microbe-derived, and endogenously produced metabolites can activate AHR in the lung, influencing local immune responses (Table 2). These include tryptophan metabolites from oral or respiratory microbiota that can cross mucosal barriers and engage AHR on different immune and epithelial cells, molecules derived from lung pathogenic bacteria (e.g., phenazines from *Pseudomonas aeruginosa* or naphthoquinones from *Mycobacterium tuberculosis*), and endogenous molecules differentially produced upon infection (e.g., increased kynurenine levels upon different viral infections).^47,252,259,263,277,325^ Consequently, in the lungs, the AHR controls detoxification pathways, mucus production (e.g., upon exposure to ligands or bacterial or viral infections), the expression of inflammatory mediators, and immune cell recruitment (e.g., in response to different infections and pollutants).^47,252,259,263,277,325–329^ In concordance, the involvement of the AHR in different lung diseases, including inflammatory, allergic, and infectious diseases (e.g., asthma, COPD, tuberculosis, COVID, and flu), as well as lung cancer, has been reported.^47,60,252,259,327,328,330–334^

In summary, the integration of environmental, microbial, and endogenous signals through receptors such as the AHR is fundamental to the function of barrier organs. The skin, lungs, and gut are not merely static barriers; they are active, dynamic interfaces where the balance between defense and tolerance is continuously maintained and, when disrupted, can lead to pathology. Through its role in mediating detoxification, cellular maintenance and differentiation, and modulating immune responses, AHR stands out as a key player in the preservation of barrier integrity. The interplay between AHR and the microbiome further underscores the complexity of these interactions. Microbial metabolites can serve as natural ligands for AHR, reinforcing barrier function and immune regulation. Conversely, dysregulated AHR signaling can contribute to pathological conditions by disrupting these delicate interactions. The organ-specific roles of AHR in barrier organs highlight its versatility and potential as a therapeutic target. Future research should continue to elucidate the precise mechanisms by which AHR integrates diverse signals to orchestrate the responses of barrier organs. Such studies will be critical for the development of targeted therapies that can correct dysfunctions at the interface of environmental exposures and immune responses, ultimately paving the way for novel approaches in the treatment of a range of chronic inflammatory, immune-mediated, and infectious diseases.

## AHR as a sensor of the chemical exposome

AHR plays a crucial role as an environmental sensor, mediating the body’s response to a wide array of exogenous and endogenous compounds. This section explores the intricate relationships among AHR, the exposome, and environmental ligands, as well as their implications for toxicology. The exposome, a term coined by Christopher Wild in 2005, encompasses the totality of environmental exposures an individual encounters from conception onward.^335^ AHR stands at the forefront of the body’s interaction with the exposome, acting as a key mediator between environmental stimuli and cellular responses. Its activation by various ligands can lead to a range of physiological and pathological outcomes, influencing development, immune function, and metabolism.

### Sources of AHR ligands

Environmental AHR ligands can be broadly categorized into several groups:

#### Persistent organic pollutants (POPs)

They are a class of highly stable environmental contaminants that can persist in the environment for long periods and accumulate in living organisms.^336^ Among them, halogenated aromatic hydrocarbons (HAHs), such as polychlorinated dibenzo-p-dioxins (PCDDs), are potent AHR activators, with TCDD being the most toxic and well studied.^337^ TCDD is formed during the combustion of organic compounds and is a highly persistent environmental toxin with a human half-life of 5–10 years.^338^ Like PCDDs, polychlorinated dibenzofurans are also formed during combustion processes and are strong AHR activators.^339^ Some persistent AHR ligands are man-made chemicals (i.e., anthropogenic), such as polychlorinated biphenyls, which were widely used in industrial applications before being banned. Some of them, specifically those with planar structures, can activate AHR, contributing to various toxic effects.^340^

#### Polycyclic aromatic hydrocarbons (PAHs)

They are a class of compounds that are also formed during the incomplete combustion of organic matter.^341^ Among them, B[a]P is a well-known carcinogen found in tobacco smoke, grilled foods, and as a result of fossil fuel combustion. 3-Methylcholanthrene is often used in research to study AHR-mediated effects. Interestingly, PAHs can activate, in some cases, other xenobiotic receptors, such as the constitutive androstane receptor (CAR), leading to the induction of a variety of xenobiotic-metabolizing enzymes.^342^

#### Other external chemicals that act as AHR ligands

A number of exogenously derived ligands, such as drugs (e.g., omeprazole), do not fall into the category of POPs or PAHs.^268^ They are likely to activate AHR transiently, as they are often metabolized by xenobiotic metabolism enzymes.

#### Naturally occurring AHR ligands

In addition to synthetic chemicals, some dietary compounds can act on or give rise to AHR ligands. For example, I3C, which is found in cruciferous vegetables such as broccoli and cabbage, binds AHR with low affinity and can be converted to 3,3’-diindolylmethane, another AHR ligand, in the acidic environment of the stomach.^343^ Several flavonoids, such as quercetin and resveratrol, which are plant-derived compounds found in fruits, vegetables, and some beverages, are also AHR ligands.^344,345^ There are also microbial ligands of the AHR that can be considered either environmental or endogenous, depending on the status of the microbiome.^280^ For example, indole-3-acetic acid and tryptamine are produced from tryptophan by gut bacteria such as *Peptostreptococcus* sp. *Lactobacillus* and *Clostridium sporogenes* and act as potent AHR agonists.^280,346^ Finally, the body can also produce AHR ligands, including kynurenine, a metabolite of tryptophan produced by the enzymes IDO/TDO,^45^ or FICZ, a compound generated by UV light-mediated degradation of tryptophan and a potent activator of AHR.^42^ See the “AHR natural ligands and physiological functions” section for more details.

**Fig. 5:**
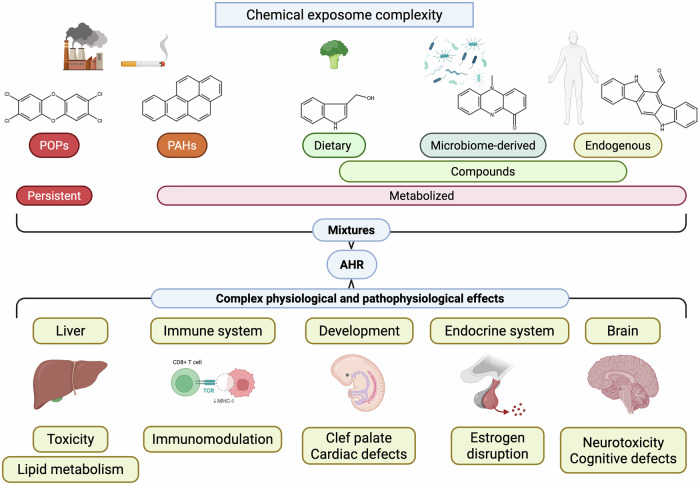
AHR as a sensor of the chemical exposome. Illustration of the complexity of the chemical exposome, encompassing persistent (e.g., POPs) and metabolized compounds of dietary, microbiome-derived, and endogenous origin. These exposures act as mixtures and may converge on the AHR, leading to diverse physiological, pathophysiological and developmental effects on key organs and systems, including the liver, immune system, endocrine system, and brain. The figure was created via BioRender

These diverse groups of AHR ligands highlight the role of the receptor as a sensor, mediating the responses of both exogenous toxic and nontoxic molecules and endogenous metabolites (Fig. 5). The AHR likely evolved primarily as a sensor for endogenous or bacterial molecules, with its role as a xenobiotic receptor emerging later to safeguard tryptophan sensing, even though this latter function was the first to be discovered. One complexity for future exposome research is therefore to consider the effects of mixtures of these different ligands.

### Toxicological effects of AHR activation

Activation of the canonical AHR pathway (see the “AHR signaling pathways and crosstalk” section) induces the expression of metabolic enzymes (Phases I, II, and III) that are generally protective but can generate toxic intermediates depending on the nature and persistence of the ligand, potentially leading to variable effects, including endocrine disruption and multiple toxicities.

#### Liver toxicity

AHR plays a significant role in mediating the toxic effects of various environmental pollutants, particularly dioxins, on the liver. For example, TCDD can lead to abnormal accumulation of lipids in liver cells.^347^ Moreover, prolonged activation of AHR by environmental compounds can result in dysregulation of the inflammatory process, potentially leading to liver inflammation.^348^ Sustained steatosis and inflammation can ultimately lead to liver fibrosis.^227,349^ Interestingly, studies using AHR knockout mice have shown that AHR deficiency is also involved in the development of liver fibrosis,^19^ suggesting that TCDD inhibits homeostasis processes in the liver and the effects of endogenous ligands, highlighting the complexity of studying the chemical exposome that activates AHR. Among the suspected mechanisms of AHR-mediated hepatotoxicity, the induction of xenobiotic metabolism enzymes can lead to oxidative stress and inflammation,^350^ and the disruption of normal lipid metabolism in the liver contributes to steatosis.^351^ This characterization is key, as understanding the mechanisms of AHR-mediated hepatotoxicity could lead to new therapeutic strategies for liver disorders.^352^

#### Immunotoxicity

Immunotoxicity is the study of the adverse effects of chemical, biological, and physical agents on the immune system, including both their suppression and inappropriate activation. One of the first demonstrated effects of AHR activation by environmental contaminants was thymic atrophy, an AHR-dependent process.^20^ AHR can also suppress antibody production and the memory response to viral infections, resulting in lower levels of virus-specific IgG.^353^ The suppression of antibody responses extends to both primary and secondary immune responses, and in human population studies, exposure to AHR-binding POPs has been associated with lower responses to vaccination.^354^ AHR-mediated effects on antibody responses likely reflect the modulation of events in multiple cell types. AHR ligands can affect B-cell differentiation both in vivo and in vitro. AHR activation also significantly impacts CD4^+^ and CD8^+^ T-cell differentiation through a combination of direct action and effects on antigen-presenting cells.^229^ For example, AHR activation with TCDD, ITE and kynurenine decreases the differentiation of CD4^+^ T cells into T follicular helper cells, a subset that is essential for high-affinity isotype-switched antibodies, *via* a process that requires AHR in T cells.^232^ In accordance with the type of ligand and immune stimulus, AHR activation can induce CD4^+^ T cells with a regulatory phenotype (Tregs) or Th17 cells, inducing the expression of Aiolos, which silences IL2 expression.^355,356^ AHR activation is also associated with the induction of CD4^+^CD8αα^+^ double-positive intraepithelial lymphocytes (DP IELs) with regulatory functions.^311^ In the latter case, indole derivatives of tryptophan, produced by Lactobacillus, promote AHR activation. More generally, these effects underscore the importance of better characterizing the specificity of each AHR ligand to understand its immunomodulatory effects.

#### Developmental toxicity

AHR activation plays a significant role in developmental toxicity, particularly affecting palate formation, kidney development, and the cardiovascular system. Indeed, TCDD exposure at gestation day 10.5 in mice inhibits palatal shelf development, resulting in cleft palate.^357^ This effect is AHR dependent, as AHR knockout mice are resistant to TCDD. Moreover, it involves interactions between AHR and retinoic acid signaling, which is another part of the endogenous metabolome.^358^ Similarly, AHR activation can cause hydronephrosis in developing animals due to hyperproliferation of epithelial cells in the ureter.^359^ The etiology involves altered expression of EGFR, EGF, and transforming growth factor-α. Another recurrent developmental effect of AHR activation due to TCDD exposure is the occurrence of cardiac abnormalities in avian, mammalian, and fish embryos.^360^ For example, in mice, early embryonic exposure to AHR agonists causes cardiotoxicity that persists into adulthood, increasing susceptibility to heart disease.^361^

#### Endocrine disruption

AHR activation plays a significant role in endocrine disruption, particularly affecting estrogen and androgen signaling as well as thyroid hormone metabolism. For example, TCDD and other AHR agonists can repress estrogen-dependent gene transcription. In that case, ligand-bound AHR can interact with unliganded ERα, forming a complex that is recruited to estrogen-responsive genes.^362^ Competition for shared coregulators such as ERAP140 and SMRT between AHR and the ER can lead to inhibitory crosstalk.^363,364^ AHR activation can alter estrogen levels by inducing estradiol-metabolizing enzymes, such as CYP1A, thereby affecting the tissue levels of estrogens.^61^ Furthermore, AHR has been shown to act as an E3 ubiquitin ligase that induces the ubiquitination and proteasome-driven degradation of steroid receptors.^103,105^ AHR activation can also disrupt thyroid hormone homeostasis and metabolism. AHR agonists such as TCDD and PCB126 have been shown to significantly downregulate the expression of sodium/iodide symporter (NIS, a membrane protein that actively transports iodide (I⁻)) in thyrocytes or interfere with the synthesis and degradation of thyroglobulin, a key process in thyroid hormone production.^365^ Exposure to AHR agonists can alter the expression of genes involved in thyroid hormone metabolism, including deiodinases and sulfotransferases.

#### Neurotoxicity

AHR activation has been associated with various neurotoxic effects, particularly those affecting cognitive function and neurotransmitter signaling. For example, in chronic kidney disease (CKD) models, cognitive impairment is correlated with serum levels of indoxyl sulfate, an AHR agonist.^366^ AHR-mediated blood‒brain barrier disruption appears to be an important mechanism involved in cognitive impairment in CKD patients.^367^ At the cellular level, excessive activation of AHR signaling through constitutive expression disrupts both the tangential and radial migration of neuronal precursors in the developing brain, which could impact cognitive function. TCDD exposure also reduces granule neuroblast survival, potentially impacting cerebellar development.^368,369^ While AHR activation is generally associated with neurotoxic effects (see above), recent research has also identified potential neuroprotective roles for AHR in certain contexts. Activating AHR with endogenous ligands, such as FICZ or I3C, has been shown to alleviate cognitive deficits in an Alzheimer’s disease mouse model by upregulating the endogenous Aβ catabolic enzyme neprilysin.^370^ Moreover, AHR activated by microbiota ligands also plays a role in maintaining the integrity and function of the gut barrier, regulating immune responses. This could modulate neuroinflammation and indirectly affect neurotransmitter signaling and cognitive function.

In conclusion, the AHR is a central molecular sensor of the chemical exposome that is capable of translating a wide array of environmental and endogenous signals into diverse biological responses. Its activation by persistent pollutants, dietary compounds, and microbiota-derived metabolites establishes a critical link between environmental exposure and key physiological processes such as metabolism, immunity, development, and neuroendocrine function. To better understand and leverage this complexity, future exposome research should prioritize the characterization of AHR ligand mixtures, the identification of selective modulators, and a deeper exploration of the microbiota–AHR axis.

## AHR in cancer

AHR activation plays important distinct roles in cancer biology even in the absence of environmental toxins.^371–375^ Depending on the context, AHR can exert either tumor-promoting or tumor-suppressive effects.^371,376^ The opposing effects of AHR in cancer likely stem from the complexity of its activation and biological functions, which are cell type-, ligand-, and context-specific.^377^ Moreover, the timing of AHR activation during tumorigenesis and its interactions with cellular signaling pathways may contribute to the complex biology of AHR in cancer.

Many tumors express high levels of AHR and produce endogenous AHR agonists, thus taking advantage of AHR activation to promote intrinsic malignant properties of tumor cells and to suppress antitumor immune responses.^63,372^ AHR drives epithelial-to-mesenchymal transition, cancer cell migration, invasion, and survival and promotes cancer stem cell characteristics.^45,173,216,378,379^ Moreover, it inhibits antitumor immunity by recruiting immunosuppressive tumor-associated macrophages, suppressing T-cell proliferation, and inducing T-cell exhaustion and death.^380–388^ AHR activation in T cells supports the differentiation of regulatory T cells and IL-10-producing type 1 regulatory T cells, both of which suppress effective antitumor responses.^176,380,387,389–392^ These findings have prompted the development of drugs that inhibit AHR or upstream AHR agonist-producing enzymes, albeit with limited success.^387,393–396^ Resistance to pharmacological inhibitors of upstream AHR agonist-producing enzymes could be due to compensation through other AHR ligand-producing pathways.^173,174,397,398^ Furthermore, cell type- or ligand-specific differences in the efficacy of AHR inhibition might hamper strategies that target AHR directly. Moreover, AHR also exerts tumor-suppressive effects and has been shown to inhibit tumor formation^399–404^ and metastasis.^334,405^ These divergent effects of AHR in cancer likely stem from the complexity of its activation and effects.

The wide variety of AHR ligands contributes significantly to its diverse effects. AHR can be activated by environmental toxins, endogenous metabolites, dietary components, and microbial products.^61,406–409^ Strong agonists such as dioxins or B[a]P, derived from pollutants, tend to induce sustained AHR activation associated with toxicity and carcinogenesis.^406^ Endogenous ligands such as tryptophan metabolites often modulate immune function and cancer cell functions, with effects dependent on ligand concentration and exposure duration.^408^ Dietary ligands such as indole-3-carbinol, which are found in cruciferous vegetables, frequently play protective roles and support detoxification and anti-inflammatory pathways.^52,410^

The metabolic activity of cancer cells is a key source of endogenous AHR ligands. Many tumors upregulate enzymes such as IDO1^176,411,412^ and TDO2,^45,386,413–415^ which catabolize tryptophan into kynurenine and downstream metabolites, AHR agonists that increase immune suppression and malignancy.^416^ In addition, tryptophan depletion increases AHR expression and activation, indicating that tryptophan stress not only impairs immune cell function but also directly amplifies AHR-mediated signaling in both tumor and immune compartments.^45,176,417^ Moreover, alternative tryptophan-catabolizing enzymes, such as the L-amino oxidase interleukin-4-induced 1 (IL4I1),^173^ contribute to the production of additional AHR ligands that are capable of modulating immune suppression and tumor progression, expanding the landscape of AHR activation beyond the classical IDO1/TDO2 pathway. Notably, a positive feedback loop exists between AHR and both basal and induced expression of the AHR agonist-generating enzymes IDO1, IDO2, TDO2, and IL4I1.^173,176,383,388,412,418–421^ IDO1/2, TDO2, and IL4I1 metabolize tryptophan into their catabolites, which activate AHR. In turn, AHR activation upregulates the expression of IDO1/2, TDO2, and IL4I1, perpetuating the cycle. This mutual reinforcement amplifies AHR signaling and the expression of these AHR agonist-producing enzymes. As a result, even transient stimulation could lead to sustained AHR activity due to the self-reinforcing nature of the feedback loop.

However, the availability of AHR ligands is a function not only of cancer metabolism but also of microbiota composition. Across multiple cancer models, microbial indole derivatives act as ligands for AHR, driving either protective or pathogenic outcomes depending on the nature of the ligand and the cellular context. In certain contexts, AHR activation is protective: in colorectal cancer, *Acinetobacter radioresistens* produces indole acetic acid, which activates AHR in intestinal stem cells, suppressing proliferation and tumor initiation *via* the Wnt–β-catenin pathway.^422^ In melanoma, *Lactobacillus reuteri* secretes indole-3-aldehyde, which activates AHR in CD8^+^ T cells to increase interferon-γ production and improve immune checkpoint inhibitor efficacy; this effect is further amplified by a tryptophan-enriched diet.^423^ Similarly, indole-3-carboxaldehyde protects against immune checkpoint inhibitor-induced colitis by activating epithelial AHR–IL-22 signaling and fostering a microbiome enriched in butyrate-producing bacteria without compromising antitumor immunity.^424^

However, inhibition of AHR can also be beneficial. In colorectal cancer, *Lactobacillus gallinarum* secretes indole-3-carboxylic acid, which inhibits the immunosuppressive kynurenine–AHR pathway, reducing regulatory T-cell infiltration and enhancing CD8^+^ T-cell responses, thereby improving anti-programmed cell death protein 1 efficacy.^425^ In pancreatic cancer, microbial metabolism of dietary tryptophan sustains AHR activity in tumor-associated macrophages, thereby promoting immune suppression; deletion or inhibition of AHR in myeloid cells reverses this effect, restoring CD8^+^ T-cell activity and enhancing the immune checkpoint inhibitor response.^384^ Extending this axis to ferroptosis, a nonapoptotic form of programmed cell death driven by iron-dependent lipid peroxidation, *Peptostreptococcus anaerobius*, which is enriched in colorectal cancer patients, produces trans-3-indoleacrylic acid, which activates AHR to increase resistance to ferroptotic cell death and promote tumor growth.^426^ These findings identify ferroptosis as a novel downstream vulnerability of the microbiota–AHR signaling pathway in cancer and suggest that the modulation of AHR activity could sensitize tumors to ferroptotic death.

Indeed, AHR has recently been implicated in the regulation of ferroptosis in cancer more generally. In prostate cancer, AHR activation by TCDD promotes ferroptosis by suppressing the nuclear receptor subfamily 4 group A member 1/stearoyl-CoA desaturase axis, increasing oxidative stress and cell death.^427^ Conversely, in lung cancer and glioblastoma, AHR functions as a ferroptosis suppressor.^428–431^ In these settings, activation of AHR, often downstream of IDO1 and kynurenine, although these can also signal independently of AHR^,^^432^ upregulates nuclear factor erythroid 2-related factor 2 and antioxidant systems, including the cystine/glutamate transporter SLC7A11, which inhibits lipid peroxidation and preserves tumor cell viability. In tumors where AHR activation protects against ferroptosis, AHR inhibition could sensitize cancer cells to this form of cell death and hence offer novel therapeutic strategies.

The molecular and cellular environments shape the function of AHR. In the GI tract, AHR, for example, maintains barrier integrity and limits inflammation, contributing to protection against colorectal cancer.^399–401^ However, AHR activation has also been shown to promote the progression of colorectal cancer.^379,425,426,433^ The timing of AHR activation may hence be another determinant of its effects. During the early stages of tumor development, AHR may play protective roles, mediating detoxification, maintaining epithelial barrier function,^46^ and suppressing inflammation.^52^ As tumors progress, sustained or dysregulated AHR activation may promote tumor aggressiveness, indicating that the effect of AHR may undergo a temporal shift from protective to protumorigenic depending on the disease stage.

AHR not only influences tumor and immune cells within the tumor microenvironment but also systemically contributes to tumor-induced hematological abnormalities in cancer patients. Tumor-released kynurenine activates AHR in megakaryocytic-erythroid progenitor cells, driving their differentiation toward megakaryocytes by inducing Runt-related transcription factor 1.^434^ This AHR-dependent mechanism contributes to the elevated platelet counts and reduced red blood cell counts observed in cancer.

In addition to its functional impact, AHR also interacts extensively with other signaling pathways. Crosstalk between AHR and EGFR involves mutual regulation of signaling pathways that influence tumor progression. As described above, AHR activation can induce EGFR phosphorylation via c-Src^115^ or promote the release of the EGFR ligands AREG and epiregulin.^435^ In non-small cell lung cancer, elevated AHR expression is associated with poor prognosis and reduced sensitivity to tyrosine kinase inhibitors; mechanistically, AHR facilitates resistance by acting as an adapter that promotes Src-mediated signaling, bypassing EGFR inhibition.^436^ In breast cancer, reactive oxygen species induce AHR activation, which enhances the expression of antioxidant enzymes and EGFR ligands such as AREG, driving tumor progression and immune cell recruitment; the combined inhibition of AHR and EGFR sensitizes these tumors to the inhibitor erlotinib.^437^ Additionally, environmental pollutants such as PAHs and dioxin-like compounds modulate this axis in a ligand-specific manner. B[a]P promotes EGFR activation and downstream AHR signaling, whereas dioxin-like polychlorinated biphenyl PCB126 binds to the extracellular domain of EGFR, inhibiting ligand-induced activation.^437^ Finally, in glioblastoma, tryptophan stress maintains the mechanistic target of rapamycin complex 1 activity via EGFR and RAS signaling, which increases AHR levels and activity, mediating increased autophagy and sustained protein synthesis under nutrient-limited conditions.^417^ These findings establish EGFR-AHR crosstalk as a key regulatory axis in cancer biology and support the development of therapeutic strategies that simultaneously target both pathways to overcome drug resistance or influence cellular responses to environmental pollutants.

Recent research has also highlighted the AHR-Src signaling axis as a driver of resistance to BRAF inhibitors (BRAFis) in melanoma, revealing novel therapeutic vulnerability.^438^ AHR is constitutively active in subsets of melanoma cells, promoting dedifferentiation and an aggressive, drug-resistant phenotype.^439^ This nongenetic reprogramming involves activation of the Src pathway and enables tumor cells to evade front-line BRAFi therapies.^438^ Importantly, targeting Src with inhibitors such as dasatinib in preclinical models resensitized BRAFi-resistant tumors, supporting a combined therapeutic approach.^438^ In parallel, resveratrol, which acts as an AHR antagonist, has shown potential in delaying resistance by suppressing AHR-driven gene expression.^439^ Additionally, vemurafenib, a widely used BRAFi, acts as an AHR antagonist in skin cells and contributes to adverse inflammatory and phototoxic responses,^440,441^ further emphasizing the central role of AHR signaling in both therapeutic resistance and side effect profiles. Together, these findings suggest that the AHR-Src axis is a promising target for improving melanoma treatment outcomes and mitigating BRAFi resistance.

**Fig. 6:**
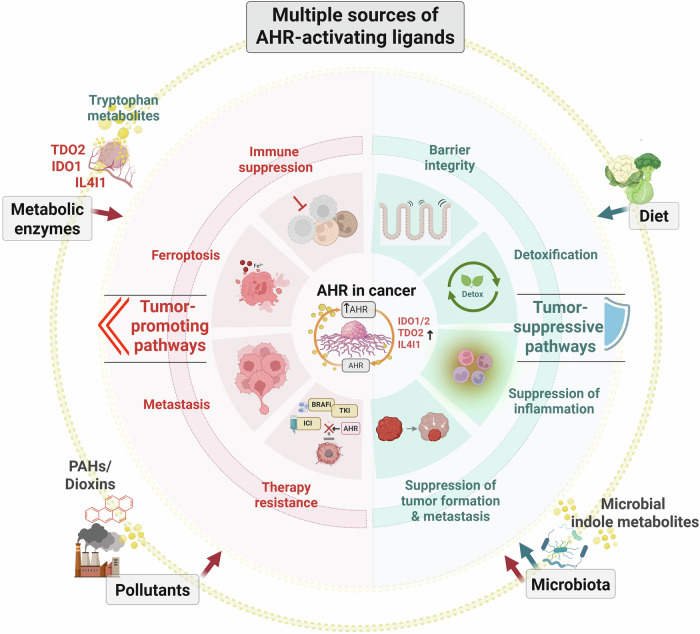
AHR in cancer. The outer circle shows the different sources of AHR-activating ligands; red arrows designate tumor-promoting effects, and green arrows indicate tumor-suppressive effects. The inner circles denote the processes affected by AHR: red, tumor-promoting; green, tumor-suppressive. The positive feedback loop between AHR and tryptophan-degrading enzymes that generate AHR agonists is shown in the center. PAH, polycyclic aromatic hydrocarbons; IDO1, indoleamine-2,3-dioxygenase 1; TDO2, tryptophan-2,3-dioxygenase; IL4I1, interleukin-4-induced 1. The figure was created via BioRender

In summary, AHR plays a highly versatile and context-sensitive role in cancer (Fig. 6). Its effects depend on the timing of activation, the specific organ and cell type, the source and type of ligands, and its interactions with other signaling pathways. Whether promoting tumor suppression or progression, AHR acts as a central hub that integrates environmental, dietary, microbial, and metabolic inputs into coordinated responses. A complete understanding of the role of AHR in cancer requires a systematic approach, considering the complexity of its signaling network and the dynamic nature of tumor evolution.

## AHR-targeting therapies

The emerging importance of AHR in immunoregulation raises the questions of the clinical translation of AHR-targeted therapies and their potential and risks. To date, only two AHR-based pharmaceuticals are available on the market, tapinarof cream 1% and benvitimod cream 1% (GSK2894512), both comprising the same active pharmaceutical component, i.e., 3,5-dihydroxy-4-isopropyl-trans-stilbene (5-[(E)-2-phenylethenyl]-2-(propan-2-yl)benzene-1,3-diol]), which was originally identified as a secondary metabolite generated by *Photorhabdus luminescens*, a bioluminescent, gram-negative bacterium that lives symbiotically within parasitic, soil-living entomopathogenic nematodes of the genus *Heterorhabditis*.^442–444^ These first-in-class drugs were approved for psoriasis therapy in China in 2019 and in the USA in 2022.^286,445,446^ All previous and current clinical trials for tapinarof are indicated in Table 3. More recently, the efficacy of tapinarof has also been shown in adult and juvenile patients with AD^59,286,446–452^ (Table 3). Therefore, in December 2024, the U.S. Food and Drug Administration (FDA) approved VTAMA^®^ (tapinarof) cream (1%) for the topical treatment of AD in adults and pediatric patients (≥2 years of age).

**Table 3:** AHR agonists under preclinical and clinical development^a^

Compound name (code)	Indication	EC_50_ [nM]	Phase of development	ClinicalTrials.gov identifier	Sponsor	Reference
Tapinarof (GSK2894512) cream (0.5% and 1%)	Plaque psoriasis in adults	13	Phase 2—dose finding (completed 2016/17)	NCT02564042	GlaxoSmithKline	^444^
Tapinarof cream 1% (DMVT-505-2002)	Plaque psoriasis in adults	13	Phase 2 (completed 2021/22)	NCT04042103	Dermavant Sciences, Inc.	^543^
**Tapinarof cream 1% (DMVT-505-3001)**	**Plaque psoriasis in adults**	**13**	**Phase 3 (PSOARING 1; completed 2022)**	**NCT03956355**	**Dermavant Sciences, Inc./IQVIA Biotech**	^544^
**Tapinarof cream 1% (DMVT-505-3002)**	**Plaque psoriasis in adults**	**13**	**Phase 3 (PSOARING 2; completed 2022)**	**NCT03983980**	**Dermavant Sciences, Inc./IQVIA Biotech**	^544^
**Tapinarof cream 1% (DMVT-505-3003)**	**Plaque psoriasis in adults**	**13**	**Phase 3—long-term extension study (PSOARING 3; completed 2022)**	**NCT04053387**	**Dermavant Sciences, Inc./IQVIA Biotech**	^545,546^
Tapinarof cream 1% (DMVT-505-3004)	Plaque psoriasis in pediatric subjects	13	Phase 3 (recruiting, estimated completion 2026)	NCT05172726	Dermavant Sciences Inc.	
VTAMA^®^ Cream 1% (tapinarof; DMVT-505-4001)	Plaque psoriasis in intertriginous areas	13	Phase 4 (completed 2024)	NCT05680740	Dermavant Sciences, Inc.	^b^
VTAMA^®^ Cream 1% (tapinarof; DMVT-505-4002)	Plaque psoriasis in the head and neck region	13	Phase 4 (completed 2024)	NCT05789576	Dermavant Sciences Inc.	^c^
Tapinarof 1% cream vs. betamethasone dipropionate 0.05% cream	Comparison of postinflammatory pigment alteration after psoriasis treatment (PIPA - Dermavant)	13	Phase 2/3 (enrolling by invitation)	NCT05981118	Wake Forest University Health Sciences	
VTAMA^®^ Cream 1% (tapinarof)	Plaque psoriasis in adults being treated with biologics	13	Phase 4 (completed 2023, results submitted 2024/25)	NCT06103695	Psoriasis Treatment Center of Central New Jersey	
Tapinarof (GSK2894512) cream (0.5% and 1%)	Atopic dermatitis	13	Phase 2—dose finding (completed 2017)	NCT02564055	GlaxoSmithKline	^452^ ^d^
Tapinarof cream 1% (DMVT-505-2104)	Extensive atopic dermatitis in pediatric subjects	13	Phase 2 (completed 2025)	NCT05186805	Dermavant Sciences, Inc.	^451^
**Tapinarof cream 1% (DMVT-505-3101)**	**Atopic Dermatitis in Children and Adults**		**Phase 3 (ADORING 1; completed 2023; results submitted 2025)**	**NCT05014568**	**Dermavant Sciences, Inc**.	^448,547^
**Tapinarof cream 1% (DMVT-505-3102)**	**Atopic Dermatitis in Children and Adults**		**Phase 3 (ADORING 2; completed 2023; results submitted 2025)**	**NCT05032859**	**Dermavant Sciences, Inc**.	^448,547^
**Tapinarof cream 1% (DMVT-505-3103)**	**Atopic dermatitis**	**13**	**Phase 3—long-term extension study (ADORING 3; completed 2024, results submitted 2025)**	**NCT05142774**	**Dermavant Sciences, Inc**.	^e^
Tapinarof cream 1% (Comparator VTAMA^®^ Cream 1%)	Plaque psoriasis in adults	13	Phase 3 (recruiting)	NCT06742957	Teva Pharmaceuticals USA/Teva Pharmaceuticals Inc.	
VTAMA^®^ Cream 1% (tapinarof)	Palmoplantar keratoderma (thickening skin layer on palms and soles) in adults	13	Phase 2 (recruiting, estimated completion 2026)	NCT06561321	Indiana University	
VTAMA^®^ Cream 1% (tapinarof)	Cutaneous lupus erythematosus	13	Early Phase 1 (not yet recruiting; estimated completion 2026)	NCT06661213	Northwestern University	
Cmpd 22 Cmpd 64 Cmpd 19 Cmpd 49	Inflammatory bowel disease	46 47 5.2 51	Preclinical	Not yet registered	Fraunhofer Cluster of Excellence Immune-mediated Diseases, Leipzig, Frankfurt/Main, Hamburg, Germany	^474^
NPD-0414-2 NPD-0414-24	Inflammatory bowel disease	- -	Preclinical	-	University of Tor Vergata, Rome, Italy	^498^
PY109 PY108	Inflammatory bowel disease	1.2 2.5	Preclinical	-	Harvard Medical School, Harvard University, Boston, MA, USA	^324^

“-” no information available

^a^Essential clinical trials are highlighted in bold. For updates, see ClinicalTrials.gov

^b^https://ichgcp.net/de/clinical-trials-registry/nct05680740

^c^https://dermsquared.com/news-research/dermavant-positive-results-from-phase-4-vtama-tapinarof-cream

^d^10.1093/bjd/ljac140.005

^e^https://www.businesswire.com/news/home/20250308653660/en/New-Analysis-of-Organon%E2%80%99s-VTAMA%C2%AE-tapinarof-cream-1-Phase-3-Data-Shows-Atopic-Dermatitis-Disease-Activity-Remained-Low-After-Treatment-Free-Interval-in-Adults-and-Children-2-Years-of-Age-and-Older

Moreover, in Japan and China, several clinical trials with naturally occurring AHR ligands derived from indigo naturalis (Qing-Dai), a traditional Chinese herbal medicine extracted and fermented from stems and leaves of *Baphicacanthus cusia* (Nees) Bremek, *Polygonum tinctorium* Ait or *Isatis indigotica* Fork, have been conducted in patients suffering from UC or psoriasis. The most prominent of these substances is indirubin, which has anti-inflammatory effects and improved symptoms but also resulted in severe adverse events, including liver dysfunction, renal dysfunction, acute colitis, colonic intussusception, and pulmonary arterial hypertension. Although the underlying mechanism of action remains unclear, AHR dependency was evidenced through *CYP 1A1* mRNA expression following 8 weeks of treatment with indirubin.^453^ However, these studies revealed some limitations in terms of sample size, ethnicity, quality and stability of the drug substance.^453^

Despite the approval and successful market launch of tapinarof and benvitimod, little information is available on the pharmaceutical development of AHR agonists for therapeutic application in IBD or other chronic-inflammatory indications.^322,454,455^ Considering the immense scientific achievements and conclusions from many preclinical studies that AHR might represent a promising therapeutic target over the last two decades,^446^ this situation is rather amazing and needs some explanation. There are two possible reasons for this phenomenon. First, in classical pharmacology, AHR activation is connected to drug metabolism because the activation of CYP1 enzymes catalyzes the oxidative biotransformation of most drugs.^456,457^ Second, there are two major categories of indications for AHR-directed therapies, i.e., inflammation vs. cancer, which require conflicting therapeutic interventions. In chronic or excessive acute inflammation, AHR agonists induce specific anti-inflammatory signaling pathways and thus may control pathological inflammatory processes and restore immune homeostasis at epithelial barriers. In contrast, in oncology, AHR-mediated immunosuppression may cause tumor progression and metastasis. Therefore, AHR-directed therapies involving agonists vs. antagonists need clear guidelines for clinical application that have not been implemented so far. Presently, this fact represents the most critical obstacle for the clinical development of AHR-targeted therapies that should be overcome very soon. In the following paragraphs, we summarize the current state of clinical development and application of AHR-directed therapies, which are divided into two main indications: inflammatory and tumor diseases.

### AHR agonists as novel therapeutics for inflammatory disorders

The crucial importance of AHR for maintaining integrity and immunological homeostasis at epithelial barriers has been intensively studied by many groups.^46,52,279^ A variety of naturally occurring AHR ligands, including xenobiotics, microbiota-derived compounds, phytochemicals, and endogenous L-tryptophan-derived photoproducts, have been reported to ameliorate symptoms in murine colitis models,^316,323,410,458–463^ murine dermatitis models,^59,302,464–467^ models of autoimmune encephalomyelitis,^247,468^ a murine model for chronic constriction of nerve injury,^469^ and a murine model for autoimmune prostatitis.^470^ Moreover, several reports have demonstrated the efficacy of AHR agonists in vitro in human cell lines, patient-derived primary cells or 3D organoid models for psoriasis,^471^ AD^466,472,473^ or chronic colitis.^474^ Notably, natural compounds that have been extensively used in AHR research as model agonists are not suitable for clinical translation because of their lack of drug-like properties, such as toxicity (e.g., TCDD, B[a]P), unfavorable PK or metabolic profiles, poor water solubility, which limits oral bioavailability (e.g., indirubin^475^), or known difficulties in terms of intellectual property rights for very interesting (unmodified) natural products (e.g., I3C, ITE).^476,477^ Moreover, the plethora of structurally unrelated AHR ligands and the poor understanding of their binding mode at the promiscuous AHR ligand binding pocket^478^ and their relation to different canonical, noncanonical or nongenomic signaling cascades in different immune cell types, such as macrophages,^479–483^ DCs,^484–486^ ILCs^313^ and T cells,^39,102,487,488^ hamper the clinical development of AHR agonists.

### AHR antagonists as novel therapeutics in oncology

Since a growing body of evidence indicates that AHR activation is involved in the proliferation, survival, migration, and invasion of tumor cells, the inhibition of AHR activation through AHR antagonists has emerged as a broadly efficient therapeutic strategy in experimental oncology. The first reports on the therapeutic efficacy of AHR antagonists were published 15 years ago. In particular, inhibitors of IDO1 and TDO2 were revealed as potential anticancer immunomodulators.^489–491^ The IDO1/TDO2-dependent immunosuppressive effects are mediated through the formation of the endogenous AHR agonist kynurenine, which targets tumor-infiltrating T cells. AHR activation through kynurenine causes immunosuppression and a tumor-promoting microenvironment in many cancer entities, representing a prominent immune escape mechanism.^492^ Therefore, pharmacological targeting of the IDO1/TDO2-AHR pathway may be a promising therapeutic strategy in immuno-oncology. For example, in animal models of melanoma, breast cancer, and colon carcinoma, depletion of kynurenine by treatment with engineered kynureninase has shown therapeutic effects when combined with checkpoint inhibitors or vaccines.^493^ The depletion of kynurenine may increase the number of effector T cells and the accumulation of CD8^+^ T cells in tumors, as well as interferon-γ secretion in the tumor microenvironment.^493^ In a cell culture model of triple-negative breast cancer (TNBC; i.e., MDA-MB-231 cells), pharmacologic inhibition or genetic attenuation of TDO2 or AHR increased cellular sensitivity to anoikis, a programmed cell death process triggered by substratum detachment that represents a critical step in the metastatic cascade, and reduced the proliferation, migration, and invasion of TNBC cells. In addition, in an MDA-MB-231-induced xenograft model in nonobese diabetic/severe combined immunodeficiency (NOD/SCID) mice, TDO2 inhibition decreased metastatic colonization in vivo.^494^ Notably, not only endogenous but also exogenous xenobiotic AHR agonists cause immune surveillance. For example, the activation of AHR through B[a]P increased PD-L1 expression in non-small cell lung cancer cells. This effect was reduced by the AHR antagonists CH223191 and ANF (alpha-naphthoflavone), supporting the idea of therapeutic inhibition of AHR signaling.^495^ As recently reported, the selective AHR inhibitor BAY 2416964 may potently inhibit AHR activation induced by either exogenous or endogenous AHR ligands. In vitro, BAY 2416964 restored immune cell function in human and mouse cells and further enhanced antigen-specific cytotoxic T-cell responses and the killing of tumor spheroids. In vivo, oral application of BAY 2416964 was well tolerated, induced a proinflammatory tumor microenvironment, and demonstrated antitumor efficacy in a syngeneic cancer mouse model (i.e., ovalbumin-expressing B16F10 melanoma).^394^ Recent clinical trials have demonstrated the therapeutic efficacy of BAY 2416964 in patients with different tumor diseases (Table 4). However, similar to AHR agonists, only a few AHR antagonists have entered clinical trials so far. Selective IDO1/TDO2 inhibitors that enhance or restore the immune response against several cancer entities (e.g., epacadostat, navoximod, BMS-986205, PF-06840003, and BAY 2416964) are being evaluated at different clinical phases^394,496,497^ (Table 4).

**Table 4:** AHR antagonists under preclinical and clinical development^a^

Compound name (code)	Indication	IC_50_ [nM]	Phase of development	Clinicaltrials.gov/EudraCT identifier	Sponsor	Reference
StemRegenin 1 (SR1)	High risk heme malignancies; hematopoietic recovery after umbilical cord blood transplantation	127	Phase 1/2 (withdrawn 2017)	NCT02765997	University of Minnesota; Novartis	^197,548,549^
BAY 2416964	Advanced solid tumors (NSCLC, HNSCC)	22	Phase 1 (completed 2024)	NCT04069026/2020-003547-28	Bayer AG	^394^^b^
IK-175 (IK-175-001; KYN-175)	Advanced solid or metastatic tumors (Urothelial carcinoma, bladder cancer)	-	Phase 1 (completed 2024)	NCT04200963	Ikena Oncology/Bristol-Myers Squibb	^c^
IK-175 (IK-175-002; KYN-175)	Resistant or recurrent head and neck squamous cell carcinoma (HNSCC)	-	Phase 1 (withdrawn 2022)	NCT05472506	Ikena Oncology/Bristol-Myers Squibb	
PX-A24590		60	Preclinical	-	Phenex Pharmaceuticals AG	^d^
KYN-101		22	Preclinical	-	Ikena Oncology	^387^

“-” no information available

^a^For updates, see ClinicalTrials.gov.

^b^https://clinicaltrials.bayer.com/study/21343/

^c^10.1200/JCO.2024.42.16_suppl.2599

^d^10.1039/d1md00015b; 10.1039/d4md00266k

### Strategies for the efficient development of new AHR agonists and antagonists for therapeutic purposes

Despite the contradicting indications of AHR agonists vs. AHR antagonists as well as the incomplete understanding of the pleiotropic role of AHR in the control of inflammation, tumorigenesis, and cancer immunity, the clinical translation of both AHR agonists and AHR antagonists has started, with hope for triumphant success in the coming years. Tremendous research achievements in immunology and oncology, as well as technological improvements, such as high-throughput (HTS) screening opportunities for cell-based XRE luciferase reporter gene assays to determine the EC_50_ or IC_50_ values of AHR agonists or antagonists, respectively, have inspired many research projects to identify and study new AHR ligands. Finally, novel AI-based docking software programs in combination with advanced databases allow better in silico modeling of receptor–ligand interactions of known and new AHR ligands. These opportunities will offer new dimensions for the study of a plethora of structurally distinctive AHR agonists, partial agonists, and orthosteric antagonists and help solve the enigma of ligand-induced conformational changes in the AHR molecular complex that determine canonical, noncanonical, or nongenomic signaling events or orthosteric inhibition of AHR signaling. Recent advances in understanding the structure‒function relationship of AHR ligands on the basis of in silico modeling^478^ and studying the structure of the human ligand-bound AHR cytosolic complex as a snapshot of the first step of AHR activation via high-resolution cryo-EM^54^ have accelerated the pharmacological development of new AHR-directed drugs. To date, the selection of candidates for further preclinical and clinical development is predominantly based on structural modifications and the optimization of natural compounds, such as indoles^324,498^ or other drugs.^466,499^ More recently, cell-based in vitro or AI-based in silico drug repurposing approaches have been shown to be successful strategies for the identification and validation of new highly efficient AHR agonists^474^ and antagonists.^500–503^ One of the major advantages of drug repurposing is the significant reduction in the time to market since pharmacological properties have already been demonstrated for other indications, and PK/PD and toxicological profiles are well known. In summary, these conditions are very favorable for the future development of further AHR-targeted therapies with many different indications.

However, there is still a knowledge gap in regard to the multiple mechanisms of action induced by structurally diverse AHR ligands in different cell types expressing AHR and in regard to their potential off-target effects. Further research will be essential to fill these gaps and clarify the actual impact of different ligands on biological systems. This knowledge will be critical for developing effective strategies to address these issues and represents a crucial precondition for the successful clinical translation of AHR agonists as well as antagonists.

## Conclusions and perspectives

The journey of AHR research reflects the dynamic and ever-evolving nature of scientific discovery. From its initial identification as a xenobiotic sensor to its status as a key player in immune regulation, development, and homeostasis, the AHR has continually surprised researchers with its diverse functions. In the future, AHR remains a subject of intense study, with potential implications for treating a wide range of diseases and understanding fundamental biological processes.

Overall, AHR is a remarkable type of environmental sensor that is capable of integrating signals from an extensive array of exogenous and endogenous ligands. This diversity of ligands, from persistent organic pollutants to dietary compounds, microbiome metabolites, and endogenous molecules, underscores the multifaceted roles of the receptor in toxicology, immunology, development, and metabolism. The complexity of AHR activation mechanisms, shaped by the chemical properties and affinities of its ligands, highlights the ability of AHR to mediate both protective and pathological responses. In this context, AHR exemplifies an evolutionary model of adaptability. Its relatively conserved nature across species reflects its ability to optimize biological functions while adapting to new, diverse environmental challenges, a testament to nature’s economic design in optimizing survival mechanisms.

The development of medicines for which AHR activation is the mode of action holds much promise. However, the broad expression of AHR across many cell types creates challenges related to off-target or unintended consequences. This approach should not automatically exclude AHR-binding compounds from consideration because there is accruing evidence that the consequences of engaging AHR are ligand-, cell type-, and tissue/organ-specific and are also likely influenced by the disease context. This points to ample opportunities for research. For example, a major open question to be answered is “why do different AHR ligands affect cellular responses in ways that are sometimes distinct, but in other instances are quite similar?” There is not yet an adequate answer to this question. We know that exposure to small molecules that bind AHR can worsen or improve disease, and AHR-dependent events may be similar or dissimilar depending on the reasons that are yet to be discovered. As a consequence of uncertainties, predicting the on- and off-target effects of AHR ligands is hampered by several interconnected unknowns, including (i) uncertain or absent absorption, distribution, metabolism, and excretion (ADME) data on many AHR ligands; (ii) a limited comparative mechanistic understanding of the myriad consequences of AHR ligands; (iii) limited structural knowledge of the AHR; and (iv) scant data to parse and predict the on-target versus secondary effects of bona fide AHR ligands (promiscuousness of some AHR ligands).

In conclusion, AHR is emerging as a promising therapeutic target for a range of inflammatory, immune, and proliferative disorders. Nevertheless, further research into its complex signaling pathways will be essential for enhancing the effectiveness of AHR-targeted therapies while mitigating potential adverse effects.
